# Probing erythrocytes as sensitive and reliable sensors of metabolic disturbances in the crosstalk between childhood obesity and insulin resistance: findings from an observational study, in vivo challenge tests, and ex vivo incubation assays

**DOI:** 10.1186/s12933-024-02395-9

**Published:** 2024-09-11

**Authors:** Álvaro González-Domínguez, Otto Savolainen, Jesús Domínguez-Riscart, Rikard Landberg, Alfonso Lechuga-Sancho, Raúl González-Domínguez

**Affiliations:** 1 Instituto de Investigación e Innovación Biomédica de Cádiz (INiBICA), Hospital Universitario Puerta del Mar, Universidad de Cádiz, Cádiz, 11009 Spain; 2 Division of Liver Diseases, Icahn School of Medicine at Mount Sinai, New York, 10029 USA; 3 Division of Food and Nutrition Science, Department of Life Sciences, Chalmers University of Technology, Gothenburg, SE-412 96 Sweden; 4 Unidad de Endocrinología Pediátrica y Diabetes, Servicio de Pediatría, Hospital Universitario Puerta del Mar, Cádiz, 11009 Spain; 5 Departamento Materno Infantil y Radiología, Facultad de Medicina, Universidad de Cádiz, Cádiz, 11009 Spain

**Keywords:** Childhood obesity, Erythrocytes, Insulin resistance, Metabolomics

## Abstract

**Background:**

Although insulin resistance (IR) is among the most frequent and pathogenically relevant complications accompanying childhood obesity, its role in modulating and exacerbating obesity pathophysiology has not yet been completely clarified.

**Methods:**

To get deeper insights into the interplay between childhood obesity and IR, we leveraged a comprehensive experimental design based on a combination of observational data, in vivo challenge tests (i.e., oral glucose tolerance test), and ex vivo assays (i.e., incubation of erythrocytes with insulin) using a population comprising children with obesity and IR, children with obesity without IR, and healthy controls, from whom plasma and erythrocyte samples were collected for subsequent metabolomics analysis.

**Results:**

Children with concomitant IR showed exacerbated metabolic disturbances in the crosstalk between endogenous, microbial, and environmental determinants, including failures in energy homeostasis, amino acid metabolism, oxidative stress, synthesis of steroid hormones and bile acids, membrane lipid composition, as well as differences in exposome-related metabolites associated with diet, exposure to endocrine disruptors, and gut microbiota. Furthermore, challenge tests and ex vivo assays revealed a deleterious impact of IR on individuals’ metabolic flexibility, as reflected in blunted capacity to regulate homeostasis in response to hyperinsulinemia, at both systemic and erythroid levels.

**Conclusions:**

Thus, we have demonstrated for the first time that metabolite alterations in erythrocytes represent reliable and sensitive biomarkers to disentangle the metabolic complexity of IR and childhood obesity. This study emphasizes the crucial need of addressing inter-individual variability factors, such as the presence of comorbidities, to obtain a more accurate understanding of obesity-related molecular mechanisms.

**Supplementary Information:**

The online version contains supplementary material available at 10.1186/s12933-024-02395-9.

## Background

Obesity is a chronic disorder primarily characterized by substantial fat accumulation. The deleterious health repercussions of excess body weight are of utmost importance among pediatric populations, as it is related to increased risk of developing non-communicable diseases, disability, and premature death during adulthood [1]. Although it is well-known to be usually accompanied by multiple complications, insulin resistance (IR) has been identified as a frequent forerunner state of typical metabolic dysfunctions underlying obesity at early ages [2]. However, complex molecular mechanisms and risk factors associated with such a multi-factorial condition have not yet been completely elucidated. For this purpose, metabolomics stands out as a suitable approach to get deeper insights into disease pathophysiology, as metabolites represent accurate phenotypic indicators of the close interplay between host metabolism and exogenous factors [3]. A number of metabolomics studies have been published on childhood obesity [4, 5], but most of them have relied on considering obesity as a homogeneous disorder, often overlooking the involvement of comorbidities. Thus, the influence of concomitant IR in the obesity-related metabolome has scarcely been explored [6, 7]. Accordingly, further research in well-characterized cohorts is crucial to deepen into the role of insulin disturbances in childhood obesity.

In metabolomics, biofluids are the most frequently employed matrices for identifying circulating disease-related markers in a minimally-invasive manner. As an alternative, the abundance and relative simplicity (i.e., lack of nuclei, mitochondria) of erythrocytes has sparked great interest in their use as cellular models of human metabolism [8]. Importantly, erythroid energy requirements are majorly dependent on glucose due to their incapacity to generate ATP through other mitochondrial pathways. Furthermore, because of their involvement in oxygen transport, the evolution has conferred red blood cells a set of powerful antioxidant systems to fight against the continuous exposure to reactive oxygen and nitrogen species (RONS) [9]. Inflammation is also known to impact erythroid metabolism and function, as pro-inflammatory cytokines may disturb iron homeostasis, ultimately leading to anemia [10]. Thus, we hypothesize that erythrocytes could serve as reliable and sensitive sensors of energy metabolism failures, oxidative stress, and chronic inflammation, which altogether represent the most relevant pathogenic events behind obesity and its comorbidities.

**Fig. 1:**
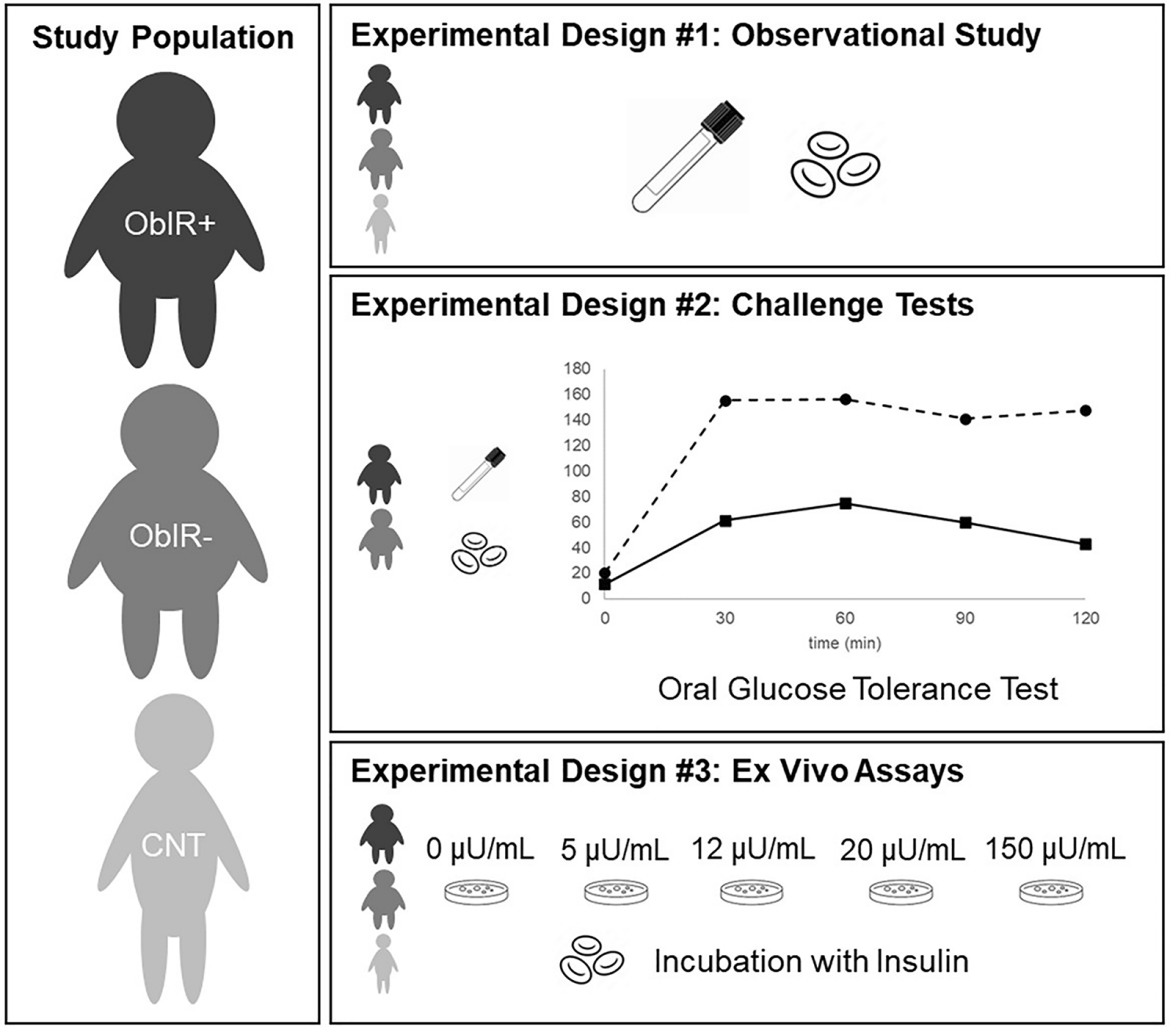
Schematic representation of the study design based on the combination of observational data, challenge tests, and ex vivo assays in a population comprising children with obesity and insulin resistance (ObIR+), children with obesity without insulin resistance (ObIR-), and healthy control children (CNT)

Herein, we sought to investigate the pivotal role that insulin-related impairments may play in modulating and exacerbating the molecular fingerprints associated with childhood obesity. To this end, we leverage a comprehensive experimental design based on the combination of observational data, in vivo challenge tests, and mechanistic ex vivo assays, as schematized in Fig. 1. The study relies on a population that comprises children with obesity, with and without IR, and healthy controls. Besides fasting examinations, the participants underwent an oral glucose tolerance test (OGTT) to assess their metabolic flexibility. Moreover, erythrocyte samples were incubated with insulin to mimic the effects of IR-related hyperinsulinemia ex vivo. Then, untargeted metabolomics was applied to plasma and erythrocytes to get a multi-compartmental characterization of the metabolic alterations occurring in the crosstalk between IR and childhood obesity.

## Methods

### Study population

A cohort of prepubertal children, aged 6 to 10 years, was recruited comprising children with obesity and IR (ObIR+, *N* = 40), children with obesity without IR (ObIR-, *N* = 24), and healthy controls (CNT, *N* = 28). This recruitment was performed within a strict protocolized clinical setting for diagnosing childhood obesity and IR, where the OGTT is solely prescribed when patients present signs of metabolic disorders. As this is an invasive and discomforting procedure, the application of an OGTT is totally discouraged in healthy people, especially in children, in accordance with the guidelines established by the Spanish Ministry of Health [11]. Obesity was defined as presenting a body mass index (BMI) over two standard deviations above the mean of the age- and sex-adjusted reference population [12], and the OGTT was used to diagnose IR according to standardized criteria [13]. Moreover, lean children who needed a blood test for routine health monitoring were enrolled as control subjects. The anthropometric characterization was performed by pediatric endocrinologists. Blood glucose, insulin, and glycated hemoglobin were measured using an Alinity automatic analyzer (Abbot, Madrid, Spain). The homeostasis model assessment of IR (HOMA-IR) was calculated using the formula: HOMA-IR = (Glc×Ins)×0.055/22.5, where Glc and Ins refer to fasting glucose (mg/dL) and insulin (µU/mL), respectively. The study was performed in accordance with the principles contained in the Declaration of Helsinki. The Ethical Committee of “Hospital Puerta del Mar” approved the study protocol (Ref. PI22/01899); all participants and/or legal guardians provided written informed consent.

### Sample collection at baseline, during the OGTT, and following ex vivo assays

From the entire study population (ObIR+, *N* = 40; ObIR-, *N* = 24; CNT, *N* = 28), fasting blood samples were collected by venipuncture using BD Vacutainer EDTA tubes (Fig. 1, observational study). Furthermore, from all children with obesity (ObIR+, *N* = 40; ObIR-, *N* = 24), additional blood samples were collected at 60 and 120 min during the OGTT (Fig. 1, challenge test). All blood samples were centrifuged at 1500 g for 10 min at 4 °C to separate the plasma. Then, the erythrocyte fraction was obtained from pellets after three cycles of washing with cold saline solution (9 g/L NaCl, 4 °C) and subsequent centrifugation at 1500 g for 5 min at 4 °C. Complementarily, aliquots of fasting erythroid samples from part of the study population (ObIR+, *N* = 24; ObIR-, *N* = 8; CNT, *N* = 11) were incubated in RPMI medium (1:1, v: v) spiked with insulin at different concentrations: (i) 0 µU/mL (negative control), (ii) 5 µU/mL (ca. average insulin levels detected in CNT), (iii) 12 µU/mL (ca. average insulin levels detected in ObIR-), (iv) 20 µU/mL (ca. average insulin levels detected in ObIR+), (v) 150 µU/mL (minimum insulin levels along the OGTT to diagnose IR) (Fig. 1, ex vivo assay). After incubation for 1 h at 37 ºC, cell suspensions were centrifuged and washed as detailed above to recover erythrocytes. The trypan blue assay was employed to corroborate cell viability along the experiments. All plasma and erythrocyte samples were aliquoted and stored at −80 °C until analysis.

### Metabolomics analysis of plasma and erythrocytes

Metabolomics analyses were performed according to the methodology described by González-Domínguez et al. (details in Supplementary Material) [14]. Afterward, raw data were preprocessed using the workflow reported elsewhere for peak picking, peak alignment, and drift correction [15], and the QC*omics* guidelines were applied for quality control assessment [16]. Annotation of metabolites was accomplished by matching experimental MS data (i.e., accurate m/z and tandem MS spectra) against those available in metabolomics databases (maximum error mass: 10 ppm), namely the Human Metabolome Database and METLIN. Furthermore, the identity of phospholipids and sulfated/glucuronidated metabolites was confirmed based on characteristic fragmentation patterns described in literature [17, 18]. To increase the confidence of annotations, a spectral library was built comprising pure standards and in-house synthetized compounds (see Supplementary Material). According to the Metabolomics Standards Initiative (MSI), four identification confidence levels were defined: level 1, confident annotation with standards; level 2, probable annotation based on databases or literature; level 3, possible annotation of compound classes; level 4, non-annotated compounds [19].

### Statistical analysis

To find differential metabolites between (i) study groups at baseline (i.e., ObIR + vs. ObIR- vs. CNT), (ii) different time points along the OGTT (i.e., 0 vs. 60 vs. 120 min), and (iii) different ex vivo concentrations of insulin (i.e., negative control vs. 5/12/20/150 µU/mL), datasets were subjected to a combination of complementary multivariate and univariate statistical tools according to a workflow well-established among the metabolomics community [20], using the MetaboAnalyst 5.0 web tool (https://www.metaboanalyst.ca/). Preliminary data processing included the removal of variables containing more than 20% missing values, kNN imputation of remaining data, removal of non-informative variables based on the interquartile range, logarithmic transformation, and Pareto scaling. Then, orthogonal partial least squares discriminant analysis (OPLS-DA) was applied to investigate the discriminant power of metabolomics data in a multivariate manner. On the basis of these models, metabolites with higher discriminant capacity were selected among those showing a Variable Importance for the Projection (VIP) parameter greater than 2. Subsequently, significance was assessed by applying univariate methods, i.e., analysis of variance (ANOVA) with Fisher LSD post hoc test and false discovery rate (FDR) correction for multiple testing. Furthermore, Pearson’s correlations were computed between significant metabolites and HOMA-IR scores.

## Results

Anthropometric and biochemical characteristics of the study population are summarized in Table 1. The participants were on average 9.1 years-old and 54.1% were male. As expected by groups’ definition, children with obesity had higher weight and BMI than healthy controls, regardless of the concomitant presence or absence of IR. Moreover, increased insulin and glycated hemoglobin levels, as well as HOMA-IR scores, were observed among ObIR + and, to a lesser extent, ObIR- subjects.

**Table 1:** Demographic, anthropometric, and biochemical data of the study population.

	CNT	ObIR-	ObIR+	*p*-value
*N*	28	24	40	–
Age (years)	8.6	8.9	9.3	NS
Sex (% male)	57.1	54.2	52.5	NS
Weight (kg)	27.4	55.7^a^	59.7^a^	3.5 × 10^−20^
Weight (Z-score)	-0.06	4.8 ^a^	5.3^a^	1.7 × 10^−19^
Body mass index (kg/m^2^)	16.6	28.2^a^	29.4^a^	5.0 × 10^−19^
Body mass index (Z-score)	−0.3	4.5^a^	4.8^a^	8.6 × 10^−21^
Glucose (mg/dL)	84.2	83.4	86.5	NS
Insulin (µU/mL)	4.8	11.3^a^	20.3^a, b^	3.2 × 10^−12^
HOMA-IR	1.0	2.4^a^	4.5^a, b^	2.8 × 10^−11^
Glycated hemoglobin (%)	5.1	5.3^a^	5.3^a^	2.3 × 10^−2^

Results are expressed as mean ± standard deviation (except for sex, expressed as percentage). Superscript letters indicate significant differences between study groups (p-value < 0.05)

^a^Denotes significant differences when compared to the control group, ^b^denotes significant differences when compared to the ObIR- group. NS, non-significant

**Fig. 2:**
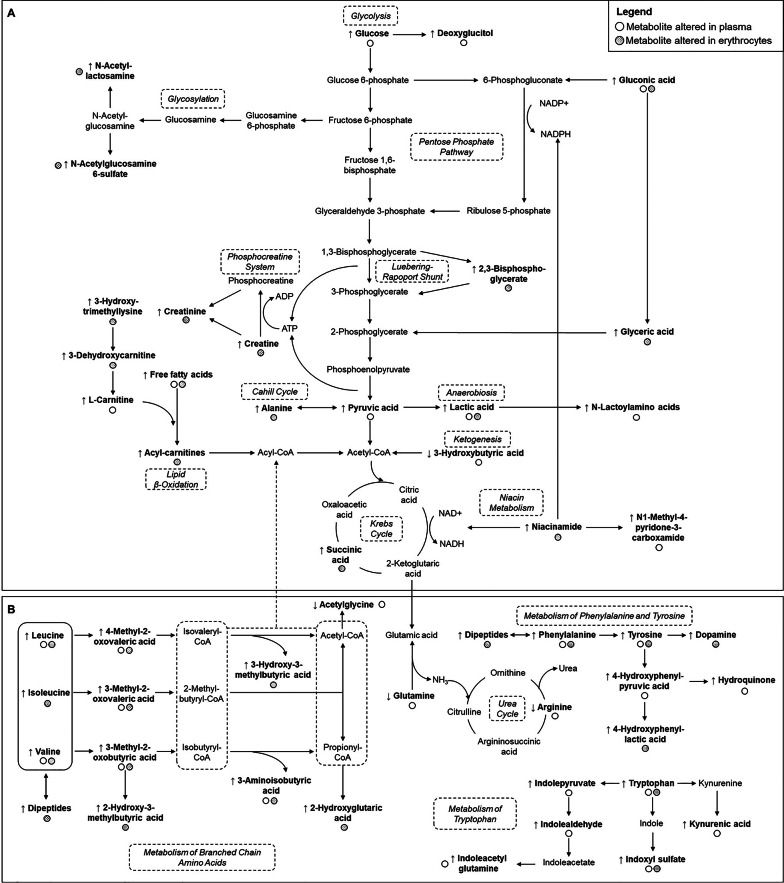
Overview of metabolite differences detected in energy-related pathways (**A**) and amino acid homeostasis (**B**)

At fasting, we identified differences in 116 plasma and 83 erythroid metabolites (Tables S1-S2). In general, levels of energy intermediates, amino acids, markers of oxidative stress and nucleotide catabolism, cholesterol derivatives, sphingolipids, and exposome-related metabolites were higher in children with obesity, whereas reduced contents were observed for 3-hydroxybutyric acid, arginine, glutamine, N-acetylglycine, pyroglutamic acid, xanthosine 5-triphosphate, fatty acid amides, and dietary compounds. Regarding phospholipids, the direction of association was found to be dependent on their fatty acid composition. Many of these differential metabolites were common to both obesity groups, although normally exacerbated among subjects presenting concomitant IR, and strongly correlated to HOMA-IR scores (Tables S1-S2). However, a few metabolites exclusively differed in ObIR + children, which could be regarded as specific markers of IR. Altogether, these results provide a comprehensive snapshot of the intertwined metabolic failures occurring in the crosstalk between IR and childhood obesity (Fig. 2).

**Fig. 3:**
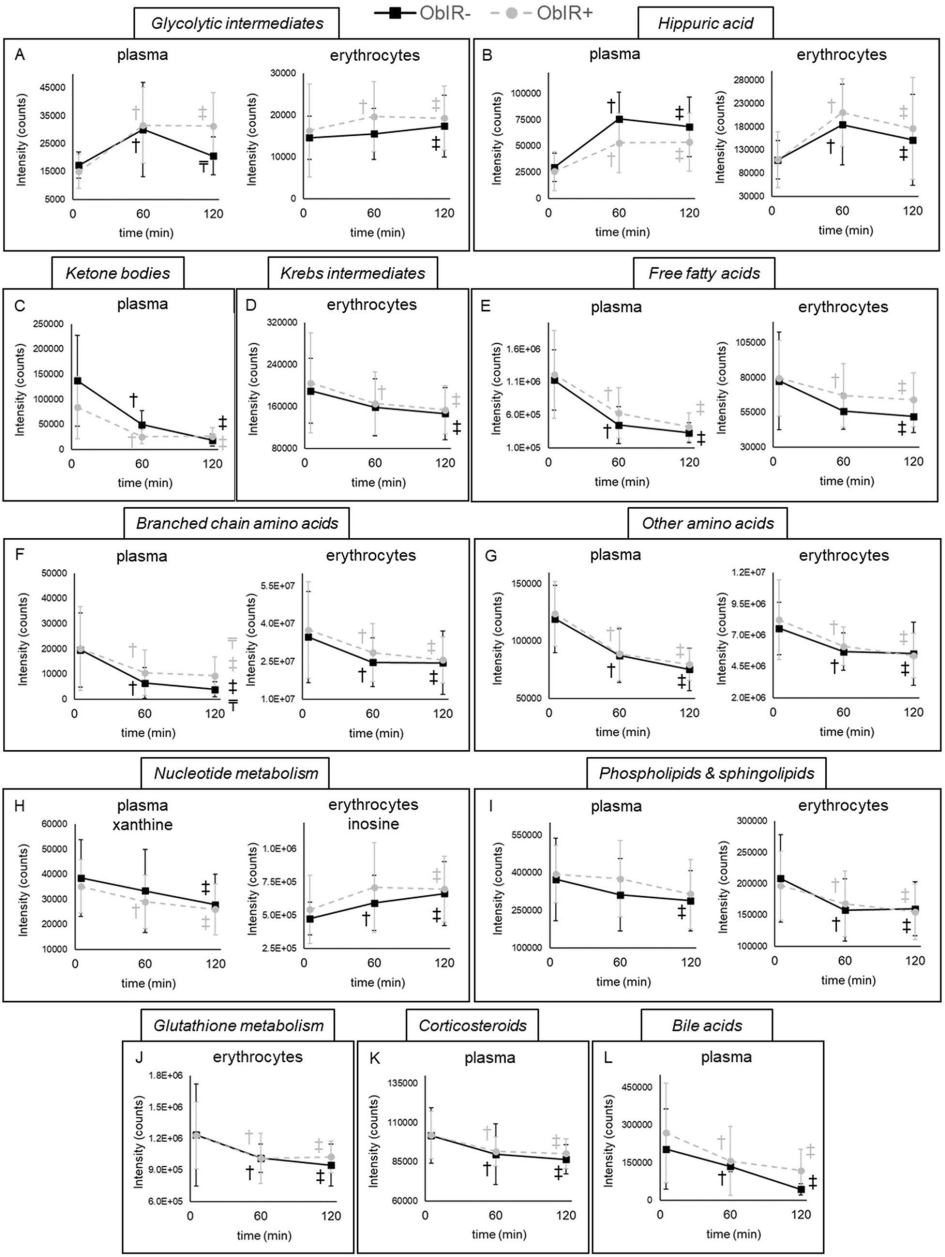
Schematic representation of time-dependent trajectories showing the effect of the oral glucose tolerance test in metabolite levels. (A) Glycolytic intermediates; (B) hippuric acid; (C) ketone bodies; (D) Krebs intermediates; (E) free fatty acids; (F) branched chain amino acids; (G) other amino acids; (H) nucleotide metabolism; (I) phospholipids and sphingolipids; (J) glutathione metabolism; (K) corticosteroids; (L) bile acids. ^†^ Denotes significant differences at 60 min when compared to the baseline (in black when differences are found within the ObIR- group, in grey when differences are found within the ObIR + group); ^‡^ denotes significant differences at 120 min when compared to the baseline (in black when differences are found within the ObIR- group, in grey when differences are found within the ObIR + group); ^₸^ denotes significant differences at 120 min when compared to 60 min (in black when differences are found within the ObIR- group, in grey when differences are found within the ObIR + group).

The OGTT provoked an increase in blood glucose and insulin at 30 min after ingestion, values that tended to decrease toward baseline levels over a 2-h time period (Figure S1). Noteworthy, the insulin peak was more pronounced among subjects with IR, and showed a slower postprandial decline compared to the ObIR- group. This was accompanied by profound metabolic alterations (Tables S3-S4), as reflected in increased levels of glycolytic and hippurate metabolites, and lower ketone bodies, succinate, acyl-carnitines, fatty and amino acids, glutathione derivatives, corticosteroids, bile acids, phospholipids, and sphingolipids. The comparison of the study groups enabled us to investigate IR-dependent metabolite trajectories, as summarized in Fig. 3.

**Fig. 4:**
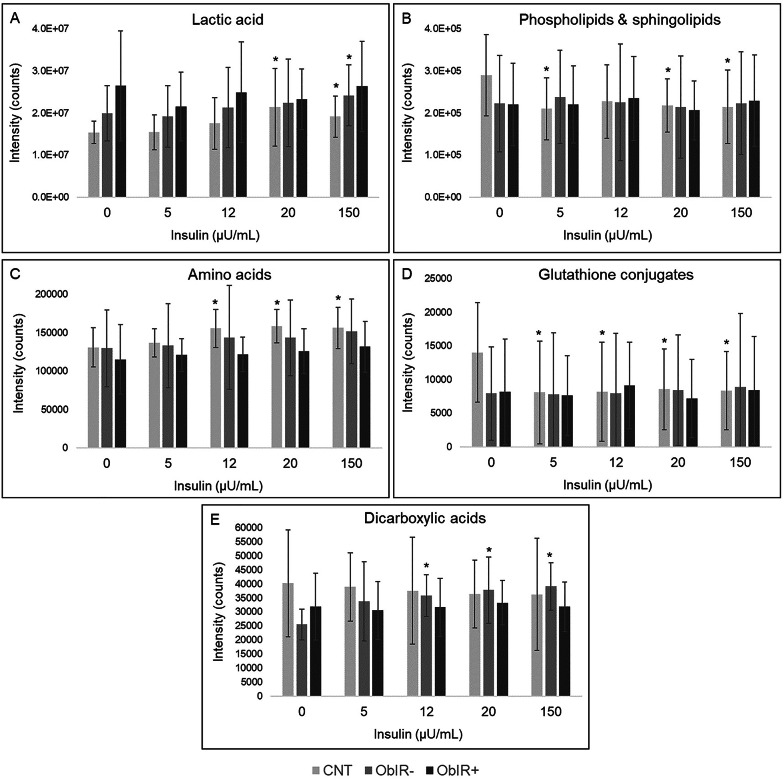
Schematic representation of the influence of ex vivo incubation with insulin in erythroid metabolite levels. (**A**) Lactic acid; (**B**) phospholipids & sphingolipids; (**C**) amino acids; (**D**) glutathione conjugates; (**E**) dicarboxylic acids. *Denotes significant differences when compared with the negative control

Finally, ex vivo assays evidenced that incubation with insulin induces similar erythroid metabolic adaptations to those observed along the OGTT (Table S5). In particular, insulin caused the over-production of lactate, amino acids, and dicarboxylic acids, as well as reductions in glutathione conjugates, phospholipids, and sphingolipids. Interestingly, most of these metabolic changes were exclusively detected in control and, to a lesser extent, ObIR- subjects (Fig. 4).

## Discussion

### Metabolic disturbances in the crosstalk between childhood obesity and insulin resistance: findings from an observational study

In congruency with excessive intake and inefficient expenditure of calories, which are considered the main hallmarks of obesity, we found altered concentrations in multiple metabolites involved in energy metabolism (Fig. 2A), at both systemic and cellular levels. This was primarily mirrored in increased plasma contents of simple sugars (e.g., glucose, deoxyglucitol), but also erythroid glycosylation intermediates (i.e., glucosamine derivatives) [21]. In line with our findings, an increment in blood pyruvate and lactate has repeatedly been described in childhood obesity [4], which could indicate boosted anaerobic glycolysis to counteract mitochondrial deficits. Interestingly, this was accompanied by over-production of N-lactoyl amino acids, which serve as biomarkers of hypoxia [22]. Besides the above-mentioned changes in glycolysis, we also detected significant perturbations in other essential pathways for erythrocyte functioning. The increase in erythroid 2,3-bisphosphoglycerate suggests impairments in the Luebering-Rapoport shunt [8]. On the other hand, the intracellular accumulation of glyceric and gluconic acids indicates disturbances in the pentose phosphate pathway (PPP), which have deleterious repercussions in oxidative control, as this pathway generates the reducing power needed to recycle glutathione [23]. This switch in energy metabolism toward the disposal of carbohydrates via glycolysis and related pathways was accompanied by altered levels of carnitine-related metabolites and free fatty acids (FFA), succinic acid, and 3-hydroxybutyric acid, indicative of dysfunctions in lipid β-oxidation, Krebs cycle, and ketogenesis, respectively [4]. Similarly, the increase observed in various metabolites participating in the phosphocreatine system (creatine, creatinine) and niacin metabolism (niacinamide, N1-methyl-4-pyridone-3-carboxamide), which are responsible for managing the energy pool (i.e., ATP, NADPH), further supports profound failures in catabolism. Noteworthy, these energy-related impairments were normally exacerbated among children with concomitant IR and showed strong correlations with HOMA-IR scores. This could primarily be allocated to the pivotal role of insulin in regulating carbohydrate and lipid metabolisms, but also to the involvement of many of these intermediates in bidirectionally modulating insulin production and signaling (e.g., influence of hyperglycemia in the development of IR, lipotoxicity induced by acyl-carnitines and FFAs) [4].

Another family of metabolites that was found to be widely disturbed in childhood obesity were amino acids and derivatives (Fig. 2B). The relationship between branched-chain amino acids (BCAAs) and obesity has been reported to involve various intertwined players, including higher dietary intake, abnormal transporter expression, IR-triggered blunted capacity of insulin to inhibit proteolysis, and overflow to skeletal muscle as a result of impaired catabolism in adipose tissue, which ultimately lead to the accumulation of BCAAs and by-products (e.g., α-ketoacids, short-chain acyl-carnitines, peptides) in blood [24]. In turn, this increment of BCAA derivatives may modulate insulin homeostasis in different ways, thereby creating a vicious cycle [25]. Firstly, it should be noted that leucine and isoleucine have been reported to be insulin secretagogues, contributing to chronic hyperinsulinemia and β-cell failure. Moreover, BCAA breakdown products have been described to cause mitochondrial dysfunction and impaired insulin signaling [25]. Among them, perturbed BCAA catabolism could be in part responsible for over-production of acyl-carnitines, thus establishing a plausible link between obesity-related impairments in amino acids and lipids. Finally, other studies have found that BCAAs also promote IR by activating the mammalian target of rapamycin (mTOR) complex. All this could explain the exacerbated increase that we found in plasma levels of BCAAs and derivatives among children with obesity and IR when compared to the ObIR- group. However, and more interestingly, the analysis of the erythroid fraction revealed that many of these BCAA-related alterations were exclusively detected in subjects presenting concomitant IR, which highlights the potential of red blood cells as reliable sensors to investigate IR-specific metabolic disturbances. A similar trend was observed in plasma and erythroid contents of aromatic amino acids (ArAAs), which could be mainly allocated to their competition with BCAAs for the same cellular transporters [24]. Furthermore, this increased bioavailability might be behind the over-production of uremic toxins (4-hydroxyphenyllactic acid, 4-hydroxyphenylpyruvic acid, indole compounds, kynurenic acid) with the involvement of pro-inflammatory gut microbiota, compounds that contribute to boost oxidative stress and inflammation [26]. Besides these increments in BCAAs and ArAAs, we also found reduced plasma levels of arginine and glutamine in children with obesity, in line with previous studies reporting disrupted nitrogen metabolism through the urea cycle and impaired production of nitric oxide [4]. On the other hand, as N-acetylglycine is generated by conjugation between glycine and acyl-coenzyme A, its reduction in plasma could be regarded as an indirect marker of impaired lipid metabolism through β-oxidation [27].

Oxidative stress is another pathogenic hallmark of obesity and related complications, derived from imbalances between RONS production and the neutralizing capacity of antioxidant systems [28]. Under this pro-oxidative scenario, obesity has been associated with profound defects in antioxidant defenses, such as the glutathione (GSH) system [29]. In the present study, this impaired GSH homeostasis was reflected in decreased plasma levels of pyroglutamic acid (GSH precursor) and increased erythroid levels of cysteinyl-glycine (GSH breakdown derivative). Furthermore, we found elevated concentrations of 2-hydroxybutyric acid in both plasma and erythrocytes, a by-product that is generated in great amounts when the body over-express the glutathionyl pathway to face oxidative stress [30]. As a consequence of this compromised antioxidant activity, our results also evidenced a substantial raise in circulating and cellular contents of numerous compounds resulting from the oxidative damage to nucleic acids (8-hydroxyguanine), proteins (3-nitrotyrosine, dityrosine), lipids (malondialdehyde, 4-hydroxynonenal, trans-2-hexenal, hexanal, 4-oxononenal glutathione), and catecholamines (leukoaminochrome, adrenochrome, adrenolutin). In this respect, we similarly found an accelerated breakdown of purines and pyrimidines in childhood obesity, with decreased content of nucleotides (xanthosine 5-triphosphate) and further generation of other catabolites (hypoxanthine, uric acid, 5-hydroxyisouric acid, allantoin, 2’-deoxyinosine, ureidosuccinic acid, N-acetylcytidine).

Metabolomics analysis of plasma and erythrocytes also revealed a close relationship between childhood obesity, IR, and multiple lipid classes. Compared to controls, obesity was associated with higher levels of plasma steroids, including androgens, estrogens, progestogens, and corticosteroids. This concurs with studies describing that excess body weight may induce the over-production of sex hormones through a complex meshwork of interrelated mechanisms, such as secretion of the gonadotropin-releasing hormone, expression of the adrenocorticotropic hormone, and reduction of sex hormone-binding globulin levels [31]. Noteworthy, most differential steroid hormones identified here were detected in their sulfated form, showed strong correlations with the HOMA-IR score, and were normally exacerbated among ObIR + subjects, in agreement with previous data suggesting that steroid sulfation is fundamental for endocrine balance [32]. Similar results were observed when studying the plasma bile acid profile, with significant increases in childhood obesity, especially among subjects presenting concomitant IR. In line with our findings, it has been reported that obesity promotes bile acid synthesis to facilitate body fat absorption and regulate insulin secretion by activating many signaling pathways [33]. Fatty acid amides participate in the endocannabinoid system, modulating food intake and energy balance through appetite-stimulating mechanisms and lipogenesis. Obesity has been related to polymorphisms in enzymes controlling this pathway [34], but scarce data is available about its influence in the levels of these endocannabinoid analogues. Herein, we found lower erythroid levels of various of these lipids, but only in the ObIR + group, suggesting a close link between IR and deficits in the endocannabinoid system. Regarding phospholipids, children with obesity showed decreased content of lyso-phospholipids and phospholipids containing polyunsaturated fatty acids (PUFAs), whereas species enriched in saturated fatty chains (i.e., myristic and palmitic acids) were found to be increased. The obesity-related reduction of lyso-phospholipids has been associated with impaired lecithin cholesterol acyltransferase activity [35]. This disruption causes diminished release of lyso-phospholipids and lowered esterification of cholesterol, which increases the bioavailability of this latter and could explain the over-production of other derivatives (i.e., steroid hormones, bile acids), as aforementioned. In contrast, the literature on the association between obesity and phospholipids is more controversial. As PUFAs are essential for membrane fluidity and permeability, we hypothesize that saturation degree imbalances could play a pivotal role in membrane destabilization processes leading to cell apoptosis. These phospholipid alterations were accompanied by increased erythroid content of various ceramides and sphingomyelins, reinforcing the pivotal role of membrane lipids in the pathogenesis of childhood obesity. Ceramides are bioactive lipids involved in the stimulation of eryptosis, an apoptosis-like death process that leads to anemia [36]. Thus, their accumulation has been reported to be a typical feature of obesity, mainly attributable to enhanced de novo synthesis due to higher availability of palmitoyl-CoA precursors, but also to increased sphingomyelinase activity [37]. Despite this exacerbated catabolism, we also detected raised contents of parent sphingomyelins, possibly as a result of simultaneous over-expression of sphingomyelin synthases [38].

To conclude, our findings also suggest the involvement of environmental factors in the development of IR and childhood obesity. We detected an inverse association between obesity and several metabolites derived from the intake of polyphenol-rich foods (e.g., methylcatechol sulfate, 2-hydroxybenzoic acid, 4-hydroxybenzaldehyde), flavanol-rich foods (e.g., 5-(3’,4’-dihydroxyphenyl)-γ-valerolactone 3’-sulfate), wholegrains (e.g., 5-aminovaleric betaine, N-(2-hydroxyphenyl)acetamide sulfate), and citrus fruits (e.g., proline betaine) [39]. In contrast, caffeine levels were found to be higher among children with obesity. This could be allocated to greater adherence to unhealthy dietary habits, often characterized by low preference for plant-based foods and increased consumption of Western diet components. In this respect, it should be noted that many of these diet-related metabolites were microbial-transformed compounds (e.g., phenolic acids, valerolactones). Besides participating in nutrient absorption, gut microbiota is involved in the synthesis of numerous bioactive metabolites, many of which have been found to be altered in the present study, including short chain organic acids (e.g., succinic acid), aromatic amino acid derivatives (e.g., indole compounds), B-group vitamins (e.g., niacinamide), and bile acids. In this sense, growing evidence suggests that obesity-related metabolic disturbances could be not only the consequence of failures in the endogenous metabolism, but also of disturbed microbial-host interactions [40]. On the other hand, children with obesity also showed increased blood levels of metabolized compounds related to the exposure to polycyclic aromatic hydrocarbons (e.g., napthtyl sulfate, 1-hydroxy-2-naphthoic acid) and parabens (e.g., propylparaben sulfate), well-known endocrine disruptors with obesogenic properties [41].

#### Influence of insulin resistance in individuals’ metabolic flexibility: findings from a challenge test

Besides fasting determinations, children with obesity underwent an OGTT to elucidate the effect of IR in individuals’ metabolic flexibility (i.e., their capacity to resolve acute metabolic stresses). The transitory hyperinsulinemia triggered by the OGTT is known to stimulate glycolysis and related pathways to manage the oversupply of carbohydrates, while blocking alternative energy sources, such as the Krebs cycle, ketogenesis, lipolysis, β-oxidation, proteolysis, and gluconeogenesis. In line with other metabolomics studies [42], we found that this is reflected in the accumulation of a variety of glycolytic metabolites and simultaneous reduction of Krebs intermediates, ketone bodies, hydroxylated and free fatty acids, acyl-carnitines, amino acids and derivatives, in both plasma and erythrocytes (Tables S3-S4). Furthermore, we observed significant increases in hippuric and 4-methylhippuric acid, compounds derived from metabolization of benzoate, a preservative present in glucose solutions administered during the OGTT. Nonetheless, the most interesting findings were obtained when comparing differential dynamics between ObIR- and ObIR + subjects. The increase in glycolytic intermediates was more pronounced among children with concomitant IR, whereas only metabolically healthy individuals were able to restore baseline levels at the end of the OGTT curve (Fig. 3). Moreover, IR was associated with blunted inhibition of ketogenesis, lipolysis, and proteolysis, as evidenced by smaller reductions in ketone bodies, FFAs, acyl-carnitines, and BCAAs. Altogether, these results support that IR may impair metabolic flexibility in children with obesity, in line with previous studies conducted in adults [43, 44]. Noteworthy, this is the first time that IR has been found to affect erythroid metabolic adaptations in response to an OGTT. These findings are of utmost relevance as demonstrate that, although glucose uptake is insulin-independent, IR may still compromise erythrocyte homeostasis.

This switch from catabolism to anabolism also affected nucleic acid metabolism, as mirrored in increased content of inosine (in erythrocytes) and decreased content of its degradation product xanthine (in plasma) [44]. In this sense, we observed a substantial reduction of phospholipids and sphingolipids in plasma and erythrocytes, changes that were more pronounced among ObIR- subjects, suggesting reduced lipid mobilization from adipose tissue as a result of insulin-induced suppression of lipolysis [45]. The OGTT was also associated with lower levels of various glutathionyl intermediates, which could be indicative of impaired GSH synthesis [46]. In line with previous studies, reductions in corticosteroids could be allocated to diurnal falls related to the circadian rhythm, rather than OGTT-induced metabolic adaptations [47]. A similar decline was found in various unconjugated bile acids, plausibly because of insulin-mediated repression of de novo synthesis and stimulation of their conjugation [48].

#### Role of insulin in modulating erythroid metabolism: findings from ex vivo assays

As a complementary approach, ex vivo assays were performed to deepen into the involvement of insulin in obesity-related erythroid disturbances, discarding potential confounding interferences derived from the complex systemic environment. The incubation with insulin primarily caused the over-production of lactic acid when compared to the negative control, indicative of boosted glucose catabolism. However, this metabolic adaptation was exclusively detected in healthy children and, to a lesser extent, ObIR- subjects, plausibly because of the insensitivity of IR-erythrocytes to respond adequately to insulin action. This concurs with our OGTT findings, further reinforcing that IR may have a profound impact on erythroid metabolism, in contrast to the traditional hypothesis that erythrocytes are insulin-independent cells. As reported in OGTT experiments, the anti-lipolytic activity of insulin was reflected in lowered phospholipids and sphingolipids, but again only in the control group. The opposite trend was found when studying the amino acid profile, with insulin inducing a raise in their contents, unlike the decline observed in the OGTT. These apparently contradictory results could be explained by the incapacity of erythrocytes to perform protein synthesis due to the lack of functional ribosomes. In the absence of systemic in vivo inputs, insulin is expected to stimulate amino acid transporters [49], thus enhancing their influx from RPMI medium and leading to their accumulation in the cytosolic space. This increase in erythroid amino acids was in general more pronounced among control individuals. Nevertheless, the comparison of both obesity groups revealed higher levels of BCAA catabolites in ObIR + children, emphasizing the close interplay between IR and BCAAs. As stated above, hyperinsulinemia also compromises GSH synthesis, which is essential to face RONS generated by oxidative catabolic pathways [46]. This lower bioavailability of free GSH could cause an impaired detoxification capacity against oxidative stressors, as reflected in decreased levels of GSH conjugates with lipid peroxidation products. In this respect, we interestingly found that insulin incubation only elicited a significant effect in healthy children’s erythrocytes, diminishing GSH conjugates toward the levels detected in negative control erythrocytes from children with obesity. Altogether, this could be allocated to exacerbated oxidative stress and impaired GSH homeostasis among ObIR- and ObIR + subjects and, therefore, the incapacity of their erythrocytes to fight against additional stress induced by extracellular insulin. To conclude, this abnormal GSH metabolism was accompanied by increased contents of various dicarboxylic acids, candidate biomarkers of fatty acid peroxidation [50]. Surprisingly, unlike the rest of metabolites reported here, these markers of oxidative stress were specific for children with obesity without IR. In general, our ex vivo assays suggest that incubation with insulin induces an obesity-like metabotype in healthy controls, as most differential metabolites experienced a change toward the levels detected in children with obesity (Fig. 4). However, metabolic inflexibility in these latter, especially when presenting concomitant IR, was found to impede erythrocytes to modulate adequately their homeostasis in response to insulin.

#### Strengths and limitations

The main strength of this study was the use of an ambitious experimental design based on the combination of observational data, nutritional challenge tests, and ex vivo assays. The recruitment of a clinically well-characterized cohort, comprising children with obesity, with and without IR, and healthy controls, enabled us investigating the close interplay between IR and obesity-related metabolic disturbances. In this respect, it should be stressed that the study cohort exclusively consisted of prepubertal children, which considerably minimized potential variability factors that could interfere our results (e.g., hormone levels, physiological IR associated to pubertal development). Then, participants underwent an OGTT to evaluate the impact of IR on phenotypic flexibility. Interestingly, we found IR to be associated with blunted capacity to inhibit ketogenesis, lipolysis, and proteolysis in response to the challenge test, and consequently with impaired metabolic health. Finally, to discard confounding variables derived from the complex systemic environment, additional ex vivo assays were conducted to focus on the effect of insulin in erythroid disruptions. Despite glucose uptake is known to be insulin-independent, we have demonstrated that IR strongly compromises energy metabolism and antioxidant defense in erythrocytes. Another added value of this work was the study of erythrocytes as a simplified model of human metabolism. Because of their intrinsic characteristics, we hypothesized that erythroid metabolite alterations could serve as reliable and sensitive biomarkers of impairments in energy homeostasis, oxidative stress, and inflammation. Furthermore, growing evidence suggests erythrocytes have higher metabolic complexity than traditionally speculated, plausibly due to the existence of cytosolic isoforms of mitochondrial enzymes and the presence of transporters capable of assimilating a myriad of compounds from the bloodstream [8]. Thus, parallel investigation of plasma and erythrocyte samples represents an exceptional approach to get a holistic and multi-compartmental characterization of metabolic alterations occurring in the crosstalk between obesity and IR. Nonetheless, some limitations deserve to be mentioned as well. The major weak point was the relatively small sample size, which makes necessary future research in larger populations to corroborate our findings. The sample size was conditioned by our strict inclusion criteria (i.e., prepubertal children) and further stratification according to the IR status, unlike most previous studies that have relied on heterogeneous populations, without considering the influence of these relevant variability factors. Moreover, as the OGTT is only performed when clinically prescribed to diagnose IR, we could not investigate its effect in control subjects.

## Conclusions

In conclusion, this study provides new insights into the close interplay between IR and childhood obesity. The application of a wide coverage metabolomics approach enabled us investigating a large number of metabolites, pinpointing to an intertwined crosstalk between the endogenous metabolism, gut microbiota, and environmental-related factors behind obesity and its comorbidities. Noteworthy, we report here for the first time the great potential of erythrocytes as cellular models to disentangle the complexity of these metabolic disorders. Altogether, these findings emphasize the crucial need of addressing inter-individual variability factors, such as the presence of concomitant IR, to obtain a more accurate understanding of disease pathophysiology in the context of precision medicine.

## Electronic supplementary material

Supplementary Material 1

## Data Availability

The datasets used and/or analysed during the current study are available from the corresponding author on reasonable request.

## Abbreviations

ArAA: Aromatic Amino Acid
BCAA: Branched Chain Amino Acid
BMI: Body Mass Index
CNT: Control Children
FFA: Free Fatty Acid
GSH: Glutathione
HOMA-IR: Homeostasis Model Assessment of Insulin Resistance
IR: Insulin Resistance
ObIR−: Children with Obesity Without Insulin Resistance
ObIR+: Children with Obesity with Insulin Resistance
OGTT: Oral Glucose Tolerance Test
RONS: Reactive Oxygen and Nitrogen Species
