# Supplementary material for: Probing erythrocytes as sensitive and reliable sensors of metabolic disturbances in the crosstalk between childhood obesity and insulin resistance: findings from an observational study, in vivo challenge tests, and ex vivo incubation assays
Source: Cardiovasc Diabetol. 2024 Sep 11;23:336. doi: 10.1186/s12933-024-02395-9 (PMC11391635; doi:10.1186/s12933-024-02395-9)
Supplement: Supplementary file 1 — Supplementary Material 1 [file 12933_2024_2395_MOESM1_ESM.docx]

**Probing erythrocytes as sensitive and reliable sensors of metabolic disturbances in the crosstalk between childhood obesity and insulin resistance: Findings from an observational study, in vivo challenge tests, and ex vivo incubation assays**

Álvaro González-Domínguez ^1,2^, Otto Savolainen ^3^, Jesús Domínguez-Riscart ^1,4^, Rikard Landberg ^5^, Alfonso Lechuga-Sancho ^1,4,6^, Raúl González-Domínguez ^1,*^

^1^ Instituto de Investigación e Innovación Biomédica de Cádiz (INiBICA), Hospital Universitario Puerta del Mar, Universidad de Cádiz. 11009 Cádiz, Spain. ^2^ Division of Liver Diseases, Icahn School of Medicine at Mount Sinai. 10029 New York, USA. ^3^ Division of Food and Nutrition Science, Department of Life Sciences, Chalmers University of Technology. SE-412 96 Gothenburg, Sweden. ^4^ Unidad de Endocrinología Pediátrica y Diabetes, Servicio de Pediatría, Hospital Universitario Puerta del Mar. 11009 Cádiz, Spain. ^5^ Departamento Materno Infantil y Radiología, Facultad de Medicina, Universidad de Cádiz. 11009 Cádiz, Spain.

*Corresponding author: [raul.gonzalez@inibica.es](mailto:raul.gonzalez@inibica.es)

**Supplementary methods**

**Metabolomics analysis of plasma and erythrocyte samples**

Metabolomics analyses were performed following the methodology described by González-Domínguez et al. [1]. First, aliquots of plasma samples (30 μL) were mixed with 200 μL of cold acetonitrile (4 °C), and vortexed for 3 min for protein precipitation. For cellular lysis, erythrocytes (20 μL) were added to 180 μL of cold extraction solvent containing methanol:acetonitrile:water (5:3:2, v:v:v), and vortexed for 30 min in cold. Then, all samples were centrifuged at 10000 g for 10 min at 4 °C, and supernatants were transferred to injection vials. Quality control (QC) samples were prepared by mixing equal aliquots of each of the samples under study and treated as explained above.

The analyses were carried out in an Agilent 1290 II UHPLC system coupled to an Agilent 6550 iFunnel QTOF-MS equipped with dual Jet Stream electrospray ion (ESI) source (Agilent Technologies, Santa Clara, CA). The chromatographic separations were performed by injecting 2 μL of sample extracts into an Acquity UPLC HSS T3 column (100 × 2.1 mm, 1.8 μm) (Waters, Milford, MA) thermostated at 45 °C. The mobile phases consisted of water (A) and methanol (B), both containing 0.04% formic acid, which were delivered at a flow rate of 0.4 mL/min by applying the following gradient program: 0-6 min, 5%-100% B; 6-10.5 min, 100% B; 10.5-10.51 min, 100%-5% B; 10.51-13 min, 5% B. Mass spectrometry (MS) detection was performed under positive and negative ionization in separate runs, by acquiring full scan spectra in the m/z range 50-1700 Da. Working MS conditions were as follows: capillary voltage, 3500 V; nozzle voltage, 300 V; fragmentor voltage, 175 V; gas temperature, 175 °C; gas flow, 12 L/min; nebulizer pressure, 45 psi; sheath gas temperature, 350 °C; sheath gas flow, 11 L/min. The study samples were analyzed in random order, whereas QCs were injected at the beginning of the sequence to equilibrate the instrument, as well as at intermittent points throughout the run to monitor system stability and to correct analytical drifts. Procedural blank samples were analyzed at the beginning and at the end on the analytical run to check for background noise and carryover.

**Synthesis of non-commercially available standards**

*Synthesis of N-lactoyl-amino acids [2]*

Prepare suspensions of 0.1 mmol amino acids (i.e., leucine, tyrosine, phenylalanine, tryptophan) in 300 µL of dry ethanol. Add 200 µL of 0.5 M sodium ethoxide in ethanol and stir for 2 h at room temperature. Then, centrifuge at 10000 g for 5 min to remove undissolved materials and take the supernatant to dryness using a vacuum concentrator. Add 0.4 mmol of ethyl lactate to the solid residue and incubate for 18 h at 80 ºC. After cooling, 200 µL of diethyl ether was added to the reaction mixture to recover N-lactoyl-amino acids as a solid material. Wash three times with 100 µL of diethyl ether, dry, and dissolve in 1 mL of 0.05 M NaOH.

*Synthesis of 5-aminovaleric acid betaine [3]*

Dissolve 0.125 mmol of 5-aminovaleric acid in 1 mL of MeOH:H2O (1:1, v:v) containing 0.75 mmol of NaOH. Add 1 mmol of iodomethane, seal the vial, and heat for 1 h at 80 ºC. Then, take the reaction mixture to dryness using a vacuum concentrator and dissolve in 1 mL of 1 M HCl. Evaporate to yield a yellow solid material and wash several times with acetone until resulting in a white powder. Dry solvent residues and dissolve in 1 mL of water.

*Synthesis of sulfated steroids [4]*

Dissolve 1 mg of individual steroids (i.e., 17-hydroxypregnenolone, dihydrotestosterone, androsterone) in 100 µL of 1,4-dioxane. Add 100 µL of a solution containing 10 mg of sulfur trioxide pyridine complex in N,N-dimethylformamide and stir for 4 h at room temperature. Then, quench the reaction with 1.5 mL of water, evaporate using a vacuum concentrator, and reconstitute in 0.5 mL of methanol:dimethylsulfoxide (1:1, v:v).

*Synthesis of 4-hydroxynonenal-glutathione conjugate [5]*

Prepare a solution containing 2 mM glutathione and 2 mM 4-hydroxynonenal in 1 mL of 50 mM phosphate buffer (pH 7.2), and incubate for 2 h at 37 ºC.

*Synthesis of dityrosine [6]*

Add 50 µL of 1.6 M HCl and 6 mL of 400 ppm KBrO_3_ to a solution of 5 mg of L-tyrosine in 1 mL of water. Cover the vials with aluminum foil and heat for 25 min at 150 ºC. After colling, take the reaction mixture to dryness using a vacuum evaporator and reconstitute in 1 mL of water.

**References**

1. González-Domínguez Á, Armeni M, Savolainen O, Lechuga-Sancho AM, Landberg R, González-Domínguez R. Untargeted Metabolomics Based on Liquid Chromatography-Mass Spectrometry for the Analysis of Plasma and Erythrocyte Samples in Childhood Obesity. In: González-Domínguez R, editor. Mass Spectrometry for Metabolomics (Methods in Molecular Biology, vol 2571). Humana; New York, NY, USA: 2023. pp. 115-122.
2. Jörres V, Keul H, Höcker H. Aminolysis of α-hydroxy acid esters with α-amino acid salts; first step in the synthesis of optically active 2,5-morpholinediones. Macromol Chem Phys 1998;199:825-833.
3. Tuomainen M, Kärkkäinen O, Leppänen J, Auriola S, Lehtonen M, Savolainen MJ, Hermansen K, Risérus U, Åkesson B, Thorsdottir I, Kolehmainen M, Uusitupa M, Poutanen K, Schwab U, Hanhineva K. Quantitative assessment of betainized compounds and associations with dietary and metabolic biomarkers in the randomized study of the healthy Nordic diet (SYSDIET). Am J Clin Nutr 2019;110:1108-1118.
4. Waller CC, McLeod MD. A simple method for the small scale synthesis and solid-phase extraction purification of steroid sulfates. Steroids 2014;92:74-80.
5. Uchida K, Stadtman ER. Selective cleavage of thioether linkage in proteins modified with 4-hydroxynonenal. Proc Natl Acad Sci U S A 1992;89:5611-5615.
6. Tilley M, Benjamin RE, Srivarin P, Tilley KA. Nonenzymatic preparative-scale synthesis of dityrosine and 3-bromotyrosine. Anal Biochem 2004;334:193-195.

**Table 1.** Differential plasma metabolites between children with obesity and insulin resistance (ObIR+), children with obesity without insulin resistance (ObIR-), and healthy control children (CNT). * Indicates significant differences according to the post-hoc Fisher LSD test (p < 0.05).

| **Metabolite** | **Mass (Da)** | **RT (min)** | **MSI level** | **p value** | **Fold changes** | | | **r HOMA-IR (p-value)** |
| --- | --- | --- | --- | --- | --- | --- | --- | --- |
|  |  |  |  |  | **ObIR+ vs CNT** | **ObIR- vs CNT** | **ObIR+ vs ObIR-** |  |
| *Glycolytic intermediates and related metabolites* | | | | | | | | |
| D-Glucose (or isomer) | 180.0634 | 0.72 | 1 | 2.8×10^-2^ | 1.07* | 1.07* | 1.00 | 0.32 (4.0×10^-3^) |
| D-Deoxyglucitol (or isomer) | 166.0841 | 0.92 | 3 | 2.1×10^-2^ | 1.29* | 1.03 | 1.26* | 0.32 (4.0×10^-3^) |
| Pyruvic acid | 88.0160 | 0.82 | 1 | 6.3×10^-3^ | 1.51* | 1.72* | 0.88 | 0.24 (3.5×10^-2^) |
| Lactic acid | 90.0317 | 0.89 | 1 | 1.3×10^-2^ | 1.32* | 1.24* | 1.06 | 0.26 (1.8×10^-2^) |
| N-Lactoyltyrosine | 253.0950 | 2.88 | 1 | 8.2×10^-3^ | 1.79* | 1.02 | 1.77* |  |
| N-Lactoylleucine | 203.1158 | 3.83 | 1 | 1.6×10^-2^ | 1.29* | 1.25* | 1.03 | 0.25 (2.8×10^-2^) |
| N-Lactoyltryptophan | 276.1110 | 3.88 | 1 | 9.1×10^-3^ | 1.51* | 1.53* | 0.99 | 0.23 (3.8×10^-2^) |
| N-Lactoylphenylalanine | 237.1001 | 3.93 | 1 | 4.9×10^-2^ | 1.20* | 1.13 | 1.06 | 0.24 (3.5×10^-2^) |
| D-Gluconic acid (or isomer) | 196.0583 | 0.71 | 1 | 4.3×10^-2^ | 1.12 | 1.23* | 0.91 | 0.25 (2.3×10^-2^) |
| N1-Methyl-4-pyridone-3-carboxamide | 152.0586 | 1.59 | 1 | 2.7×10^-2^ | 1.19* | 1.20* | 1.00 | 0.26 (2.1×10^-2^) |
| *Ketone bodies* | | | | | | | | |
| 3-Hydroxybutyric acid | 104.0473 | 1.46 | 1 | 1.6×10^-2^ | 0.57* | 0.82 | 0.70 | -0.42 (1.3×10^-4^) |
| *Carnitine-related metabolites* | | | | | | | | |
| L-Carnitine | 161.1052 | 0.67 | 1 | 4.9×10^-2^ | 1.14* | 1.12 | 1.02 |  |
| *Free fatty acids* | | | | | | | | |
| Dihydroxyoctadecenoic acid (DiHOME) | 314.2417 | 6.95 | 3 | 4.9×10^-3^ | 1.81* | 1.43 | 1.26 |  |
| Linoleic acid | 280.2402 | 7.10 | 1 | 4.2×10^-2^ | 1.90* | 1.24 | 1.53 |  |
| *Branched chain amino acids and derivatives* | | | | | | | | |
| L-Valine | 117.0790 | 0.87 | 1 | 1.8×10^-2^ | 1.21* | 1.30* | 0.93 | 0.26 (1.9×10^-2^) |
| L-Leucine | 131.0946 | 1.50 | 1 | 2.2×10^-2^ | 1.24* | 1.32* | 0.94 | 0.34 (2.4×10^-3^) |
| 3-Aminoisobutyric acid | 103.0633 | 0.71 | 1 | 5.6×10^-3^ | 1.28* | 1.50* | 0.85 | 0.21 (6.2×10^-2^) |
| 3-Methyl-2-oxobutyric acid | 116.0473 | 1.84 | 1 | 3.2×10^-2^ | 1.15 | 1.49* | 0.77 |  |
| 3-Methyl-2-oxovaleric acid | 130.0629 | 2.77 | 1 | 1.4×10^-3^ | 1.57* | 1.44* | 1.09 | 0.29 (9.2×10^-3^) |
| 4-Methyl-2-oxovaleric acid | 130.0629 | 2.93 | 1 | 2.8×10^-2^ | 1.33* | 1.52* | 0.87 |  |
| *Aromatic amino acids and derivatives* | | | | | | | | |
| L-Tyrosine | 181.0738 | 1.29 | 1 | 6.3×10^-3^ | 1.24* | 1.19* | 1.04 | 0.46 (2.1×10^-5^) |
| L-Phenylalanine | 165.0790 | 2.01 | 1 | 4.3×10^-2^ | 1.39* | 1.48* | 0.94 |  |
| L-Tryptophan | 204.0899 | 2.44 | 1 | 4.9×10^-2^ | 1.40* | 1.27 | 1.10 | 0.26 (2.1×10^-2^) |
| Hydroquinone | 110.0367 | 1.67 | 2 | 4.8×10^-2^ | 1.11* | 1.09* | 1.01 |  |
| 4-Hydroxyphenylpyruvic acid | 180.0423 | 2.06 | 1 | 1.4×10^-2^ | 1.36* | 1.30* | 1.04 | 0.27 (1.6×10^-2^) |
| Indoxyl sulfate | 213.0096 | 2.42 | 1 | 4.3×10^-2^ | 1.30* | 0.88 | 1.48* | 0.29 (8.6×10^-3^) |
| Indoxyl sulfate isomer | 213.0096 | 2.69 | 3 | 3.1×10^-2^ | 1.24* | 0.78 | 1.59* | 0.26 (1.8×10^-2^) |
| Kynurenic acid | 189.0426 | 2.82 | 1 | 2.9×10^-2^ | 1.67* | 1.98* | 0.85 | 0.26 (1.9×10^-2^) |
| Indoleacetyl glutamine | 303.1219 | 3.06 | 2 | 1.4×10^-2^ | 1.79* | 1.74* | 1.03 | 0.24 (2.9×10^-2^) |
| Indole-3-pyruvic acid | 203.0582 | 3.30 | 1 | 4.9×10^-2^ | 1.50* | 1.11 | 1.35 | 0.21 (6.8×10^-2^) |
| Indolealdehyde | 145.0528 | 3.30 | 3 | 3.1×10^-2^ | 1.58* | 1.35 | 1.18 | 0.23 (3.9×10^-2^) |
| *Other amino acids* | | | | | | | | |
| L-Arginine | 174.1117 | 0.63 | 1 | 4.9×10^-2^ | 0.85* | 0.82* | 1.03 |  |
| L-Glutamine | 146.0691 | 0.66 | 1 | 1.7×10^-2^ | 0.77* | 0.83 | 0.93 |  |
| N-Acetylglycine | 117.0426 | 0.88 | 1 | 7.6×10^-3^ | 0.50* | 0.80 | 0.63* | -0.37 (7.5×10^-4^) |
| Glutamyl-aspartic acid (or isomer) | 262.0801 | 0.87 | 3 | 3.1×10^-2^ | 1.25* | 1.33* | 0.94 | 0.21 (5.7×10^-2^) |
| *Oxidative stress markers* | | | | | | | | |
| Pyroglutamic acid | 129.0426 | 0.89 | 1 | 4.3×10^-2^ | 0.76* | 1.19 | 0.64* |  |
| 2-Hydroxybutyric acid | 104.0473 | 1.70 | 1 | 2.1×10^-2^ | 1.59* | 2.08* | 0.76 |  |
| Malondialdehyde | 72.0211 | 0.89 | 1 | 3.1×10^-2^ | 1.91* | 1.89* | 1.01 | 0.29 (9.5×10^-3^) |
| 4-Hydroxynonenal | 156.1150 | 3.83 | 1 | 3.0×10^-2^ | 1.07* | 1.08* | 0.98 | 0.20 (7.7×10^-2^) |
| trans-2-Hexenal | 98.0732 | 4.06 | 1 | 4.4×10^-2^ | 1.24* | 1.38* | 0.90 |  |
| Hexanal | 100.0888 | 4.45 | 1 | 3.4×10^-2^ | 1.25* | 1.44* | 0.87 |  |
| *Nucleotide metabolism* | | | | | | | | |
| Xanthosine 5-triphosphate | 523.9746 | 0.70 | 2 | 1.2×10^-3^ | 0.85* | 0.86* | 0.99 |  |
| Hypoxanthine | 136.0385 | 1.21 | 1 | 2.1×10^-2^ | 1.38* | 1.40* | 0.98 |  |
| Uric acid | 168.0283 | 0.88 | 1 | 1.1×10^-2^ | 1.61* | 1.48* | 1.09 |  |
| 5-Hydroxyisouric acid | 184.0232 | 0.89 | 2 | 8.6×10^-3^ | 1.27* | 1.17 | 1.08 |  |
| N-Acetylcytidine | 285.0961 | 2.05 | 2 | 3.9×10^-2^ | 1.33* | 1.30* | 1.02 |  |
| *Steroid hormones* | | | | | | | | |
| Pregnenolone sulfate | 396.1970 | 5.79 | 1 | 2.4×10^-2^ | 1.52* | 1.10 | 1.39 | 0.26 (1.8×10^-2^) |
| 17-Hydroxypregnenolone sulfate | 412.1920 | 5.39 | 1 | 3.7×10^-4^ | 2.25* | 1.62* | 1.39 | 0.38 (6.0×10^-4^) |
| Hydroxypregnenolone sulfate isomer | 412.1920 | 4.91 | 3 | 1.6×10^-4^ | 1.76* | 1.07 | 1.65* | 0.42 (1.2×10^-4^) |
| Hydroxypregnenolone disulfate | 492.1487 | 4.56 | 3 | 4.9×10^-2^ | 1.48* | 1.06 | 1.40 | 0.21 (5.8×10^-2^) |
| Dihydroxypregnenanedione glucuronide (isomer 1) | 524.2650 | 5.11 | 3 | 3.0×10^-2^ | 1.35* | 1.77* | 0.76 |  |
| Dihydroxypregnenanedione glucuronide (isomer 2) | 524.2650 | 5.37 | 3 | 2.4×10^-3^ | 2.07* | 2.76* | 0.75 | 0.26 (1.8×10^-2^) |
| Pregnanolone sulfate | 398.2127 | 5.65 | 3 | 4.5×10^-3^ | 1.81* | 1.28 | 1.42 | 0.40 (2.6×10^-4^) |
| Pregnanolone disulfate | 478.1695 | 4.62 | 3 | 4.9×10^-2^ | 1.50* | 0.93 | 1.62* |  |
| Pregnanetriol sulfate (isomer 1) | 416.2232 | 5.47 | 3 | 3.8×10^-2^ | 1.36* | 0.75 | 1.80* | 0.23 (4.3×10^-2^) |
| Pregnanetriol sulfate (isomer 2) | 416.2232 | 5.76 | 3 | 4.3×10^-2^ | 1.71* | 1.23 | 1.39 | 0.24 (3.2×10^-2^) |
| Pregnanetriol sulfate (isomer 3) | 416.2232 | 5.88 | 3 | 2.4×10^-3^ | 1.78* | 1.21 | 1.47* | 0.43 (5.7×10^-5^) |
| Pregnanetriol sulfate (isomer 4) | 416.2232 | 5.97 | 3 | 3.1×10^-2^ | 3.99* | 0.39 | 10.16* | 0.30 (6.2×10^-3^) |
| Dehydroepiandrosterone sulfate | 368.1657 | 5.24 | 1 | 1.9×10^-4^ | 2.42* | 1.50 | 1.61* | 0.45 (2.5×10^-5^) |
| Testosterone / Androstanedione sulfate (isomer 1) | 368.1657 | 5.48 | 3 | 1.3×10^-2^ | 13.20* | 1.23 | 10.73 | 0.25 (2.6×10^-2^) |
| Testosterone / Androstanedione sulfate (isomer 2) | 368.1657 | 5.67 | 3 | 1.9×10^-3^ | 2.01* | 1.34 | 1.50* | 0.43 (7.8×10^-5^) |
| Hydroxydehydroepiandrosterone sulfate (isomer 1) | 384.1607 | 4.25 | 3 | 3.2×10^-2^ | 1.52* | 0.90 | 1.68* | 0.25 (2.7×10^-2^) |
| Hydroxydehydroepiandrosterone sulfate (isomer 2) | 384.1607 | 4.61 | 3 | 1.6×10^-2^ | 2.17* | 1.05 | 2.06* | 0.44 (5.1×10^-5^) |
| Androstenediol sulfate (isomer 1) | 370.1814 | 5.03 | 3 | 3.1×10^-7^ | 4.10* | 2.44* | 1.68* | 0.52 (8.2×10^-7^) |
| Androstenediol sulfate (isomer 2) | 370.1814 | 5.95 | 3 | 1.9×10^-4^ | 2.10* | 1.39 | 1.51* | 0.42 (1.0×10^-4^) |
| Dihydrotestosterone sulfate | 370.1814 | 5.38 | 1 | 1.6×10^-2^ | 1.59* | 0.79 | 2.00* | 0.41 (1.9×10^-4^) |
| Androsterone sulfate | 370.1814 | 5.67 | 1 | 9.9×10^-3^ | 1.82* | 0.93 | 1.95* | 0.40 (3.0×10^-4^) |
| Androstenediol / Dihydrotestosterone / Androsterone disulfate (isomer 1) | 450.1382 | 4.35 | 3 | 3.8×10^-7^ | 3.01* | 2.15* | 1.40* | 0.45 (2.7×10^-5^) |
| Androstenediol / Dihydrotestosterone / Androsterone disulfate (isomer 2) | 450.1382 | 4.71 | 3 | 1.3×10^-4^ | 2.24* | 1.67* | 1.34 | 0.46 (1.4×10^-5^) |
| Androstenediol / Dihydrotestosterone / Androsterone glucuronide | 466.2567 | 5.83 | 3 | 5.0×10^-3^ | 2.53* | 1.44 | 1.76 | 0.43 (8.2×10^-5^) |
| Androstanediol / Dihydroandrosterone sulfate (isomer 1) | 372.1970 | 5.20 | 3 | 4.2×10^-4^ | 2.49* | 1.20 | 2.08* | 0.33 (2.4×10^-3^) |
| Androstanediol / Dihydroandrosterone sulfate (isomer 2) | 372.1970 | 5.52 | 3 | 1.2×10^-5^ | 2.70* | 1.42 | 1.90* | 0.43 (7.9×10^-5^) |
| Androstanediol / Dihydroandrosterone sulfate (isomer 3) | 372.1970 | 5.88 | 3 | 4.2×10^-2^ | 6.58* | 2.78 | 2.37 |  |
| Androstanediol / Dihydroandrosterone disulfate | 452.1538 | 4.51 | 3 | 4.2×10^-4^ | 2.72* | 1.60* | 1.70 | 0.41 (1.4×10^-4^) |
| Androstenetriol / Hydroxyandrosterone sulfate | 386.1763 | 4.28 | 3 | 8.6×10^-3^ | 2.00* | 1.18 | 1.69* | 0.30 (6.5×10^-3^) |
| Androstenol glucuronide | 450.2646 | 6.66 | 3 | 2.2×10^-5^ | 2.69* | 1.74* | 1.54* | 0.54 (2.0×10^-7^) |
| Estradiol glucuronide | 448.2097 | 5.24 | 3 | 1.9×10^-2^ | 1.16* | 1.06 | 1.10* | 0.47 (1.4×10^-5^) |
| Hydroxycortisol | 378.2042 | 5.92 | 3 | 2.1×10^-2^ | 1.36* | 1.77* | 0.77 |  |
| Tetrahydrocortisol | 366.2406 | 5.39 | 1 | 6.7×10^-3^ | 1.35* | 1.20 | 1.13 | 0.33 (2.6×10^-3^) |
| Dihydrocortisol / Tetrahydrocortisone / Tetrahydroaldosterone glucuronide | 540.2571 | 5.20 | 3 | 1.3×10^-4^ | 2.14* | 2.32* | 0.92 | 0.38 (4.5×10^-4^) |
| Cortolone / Tetrahydrocortisol glucuronide (isomer 1) | 542.2727 | 5.13 | 3 | 1.2×10^-3^ | 1.78* | 1.83* | 0.97 | 0.34 (2.0×10^-3^) |
| Cortolone / Tetrahydrocortisol glucuronide (isomer 2) | 542.2727 | 5.22 | 3 | 7.6×10^-6^ | 2.52* | 2.74* | 0.92 | 0.41 (1.5×10^-4^) |
| Tetrahydrodeoxycorticosterone disulfate | 494.1644 | 4.12 | 3 | 1.4×10^-3^ | 2.16* | 1.96* | 1.10 | 0.32 (3.3×10^-3^) |
| Tetrahydrodeoxycorticosterone sulfate (isomer 1) | 414.2076 | 4.74 | 3 | 7.2×10^-3^ | 1.69* | 1.11 | 1.51* | 0.32 (4.0×10^-3^) |
| Tetrahydrodeoxycorticosterone sulfate (isomer 2) | 414.2076 | 5.32 | 3 | 3.6×10^-6^ | 2.55* | 1.77* | 1.44* | 0.50 (4.1×10^-6^) |
| Tetrahydrodeoxycorticosterone glucuronide | 510.2857 | 5.99 | 3 | 2.1×10^-2^ | 1.43* | 1.46* | 0.98 | 0.27 (1.5×10^-2^) |
| *Bile acids* | | | | | | | | |
| Cholic acid | 408.2876 | 6.30 | 1 | 3.7×10^-3^ | 2.60* | 1.87* | 1.39 | 0.38 (5.4×10^-4^) |
| Cholic acid isomer | 408.2876 | 5.92 | 3 | 6.0×10^-3^ | 2.25* | 1.73 | 1.30 | 0.40 (2.2×10^-4^) |
| Deoxycholic acid | 392.2927 | 6.60 | 1 | 1.9×10^-4^ | 2.30* | 1.64 | 1.40* | 0.51 (1.4×10^-6^) |
| Deoxycholic acid isomer | 392.2927 | 6.30 | 3 | 1.0×10^-2^ | 1.80* | 1.10 | 1.64* | 0.34 (2.3×10^-3^) |
| Taurochenodeoxycholic acid | 499.2968 | 6.40 | 1 | 3.1×10^-2^ | 2.14* | 4.04* | 0.53 |  |
| Ketodeoxycholic / Nutriacholic acid sulfate (isomer 1) | 470.2338 | 5.59 | 3 | 2.8×10^-2^ | 1.28* | 1.54* | 0.83 | 0.28 (1.3×10^-2^) |
| Ketodeoxycholic / Nutriacholic acid sulfate (isomer 2) | 470.2338 | 5.68 | 3 | 2.1×10^-2^ | 1.90* | 1.60* | 1.19 | 0.37 (7.2×10^-4^) |
| 12α-Hydroxy-3-oxocholadienic acid | 386.2457 | 5.82 | 2 | 1.4×10^-9^ | 3.73* | 2.71* | 1.38 | 0.58 (2.4×10^-8^) |
| Cholestanepentol glucuronide | 628.3851 | 6.18 | 3 | 1.3×10^-2^ | 1.57* | 1.80* | 0.87 | 0.30 (6.0×10^-3^) |
| Dihydroxycholestenoic acid | 432.3240 | 6.58 | 3 | 4.9×10^-6^ | 1.64* | 1.26* | 1.30* | 0.43 (7.6×10^-5^) |
| Hydroxyoxocholestenoic acid | 430.3083 | 6.64 | 3 | 3.4×10^-2^ | 1.20* | 0.99 | 1.22* |  |
| Hydroxycholestenone | 400.3341 | 7.14 | 3 | 4.5×10^-2^ | 1.50* | 1.29 | 1.16 |  |
| *Phospholipids* | | | | | | | | |
| LPC(18:2) (isomer 1) | 519.3325 | 6.75 | 3 | 1.4×10^-2^ | 0.86* | 0.93 | 0.93 | -0.24 (3.7×10^-2^) |
| LPC(18:2) (isomer 2) | 519.3325 | 6.91 | 3 | 8.2×10^-3^ | 0.81* | 0.85* | 0.95 | -0.24 (3.4×10^-2^) |
| LPC(22:6) | 567.3325 | 6.86 | 3 | 3.1×10^-2^ | 0.85* | 0.82* | 1.03 | -0.31 (5.7×10^-3^) |
| LPC(20:4) | 543.3325 | 6.89 | 3 | 3.6×10^-2^ | 0.77* | 0.88 | 0.87 | -0.21 (5.9×10^-2^) |
| LPE(18:1) | 479.3012 | 6.91 | 3 | 3.7×10^-3^ | 0.78* | 0.77* | 1.01 | -0.23 (4.4×10^-2^) |
| LPC(22:5) | 569.3481 | 6.95 | 3 | 2.8×10^-2^ | 0.69* | 0.76* | 0.91 |  |
| LPC(18:1) | 521.3481 | 7.15 | 3 | 1.6×10^-3^ | 0.62* | 0.71* | 0.88 | -0.42 (1.0×10^-4^) |
| PC(20:4/20:4) | 829.5622 | 8.58 | 3 | 6.7×10^-3^ | 0.56* | 0.50* | 1.11 | -0.31 (4.5×10^-3^) |
| PC(15:0/22:6) | 791.5465 | 8.58 | 3 | 3.6×10^-2^ | 0.58* | 0.66 | 0.87 |  |
| PC(18:1/22:6) | 831.5778 | 8.68 | 3 | 1.4×10^-2^ | 0.50* | 1.05 | 0.47* | -0.24 (3.5×10^-2^) |
| PE(P-18:1/22:6) | 773.5359 | 8.91 | 3 | 1.5×10^-2^ | 0.67* | 0.42* | 1.61 |  |
| *Diet-related metabolites* | | | | | | | | |
| 5-Aminovaleric acid betaine | 159.1259 | 0.76 | 1 | 2.1×10^-2^ | 0.59* | 0.88 | 0.67* | -0.22 (5.8×10^-2^) |
| 5-(3',4'-Dihydroxyphenyl)-γ-valerolactone 3'-sulfate | 288.0304 | 2.68 | 1 | 2.1×10^-2^ | 0.56* | 0.29* | 1.93 |  |
| Methylcatechol sulfate | 204.0092 | 2.88 | 3 | 4.9×10^-2^ | 0.66* | 0.40* | 1.68 |  |
| N-(2-Hydroxyphenyl)acetamide sulfate | 231.0201 | 2.89 | 2 | 1.9×10^-2^ | 0.42* | 0.21* | 1.98 |  |
| 2-Hydroxybenzoic acid | 138.0317 | 4.15 | 1 | 3.2×10^-2^ | 0.59* | 0.66 | 0.89 | -0.24 (3.4×10^-2^) |
| *Exposome-related metabolites* | | | | | | | | |
| Napthtyl sulfate | 224.0143 | 3.96 | 3 | 1.9×10^-2^ | 2.05* | 1.48* | 1.39 |  |
| Propylparaben sulfate | 259.0282 | 4.02 | 2 | 4.3×10^-2^ | 2.09* | 0.91 | 2.29* |  |

**Table 2.** Differential erythroid metabolites between children with obesity and insulin resistance (ObIR+), children with obesity without insulin resistance (ObIR-), and healthy control children (CNT). * Indicates significant differences according to the post-hoc Fisher LSD test (p < 0.05).

| **Metabolite** | **Mass (Da)** | **RT (min)** | **MSI level** | **p value** | **Fold changes** | | | **r HOMA-IR (p-value)** |
| --- | --- | --- | --- | --- | --- | --- | --- | --- |
|  |  |  |  |  | **ObIR+ vs CNT** | **ObIR- vs CNT** | **ObIR+ vs ObIR-** |  |
| *Glycolytic intermediates and related metabolites* | | | | | | | | |
| N-Acetyl-D-glucosamine sulfate (or isomer) | 301.0468 | 0.73 | 3 | 4.4×10^-2^ | 1.33* | 1.03 | 1.29* |  |
| N-Acetyl-lactosamine | 383.1428 | 0.73 | 1 | 4.4×10^-2^ | 1.39* | 1.52* | 0.91 | 0.31 (2.0×10^-2^) |
| Lactic acid | 90.0317 | 0.94 | 1 | 4.4×10^-2^ | 1.43* | 1.05 | 1.36 |  |
| Glyceric acid 2,3-bisphosphate | 265.9593 | 0.87 | 1 | 3.8×10^-2^ | 1.46* | 1.31* | 1.11 | 0.41 (1.9×10^-3^) |
| Glyceric acid | 106.0266 | 0.73 | 1 | 4.4×10^-2^ | 2.04* | 1.50 | 1.35 | 0.28 (3.4×10^-2^) |
| D-Gluconic acid (or isomer) | 196.0583 | 0.70 | 1 | 4.9×10^-2^ | 1.35* | 1.22 | 1.11 | 0.29 (2.6×10^-2^) |
| Creatinine | 113.0589 | 0.71 | 1 | 4.4×10^-2^ | 1.46* | 1.32 | 1.10 |  |
| Creatine | 131.0695 | 0.72 | 1 | 4.0×10^-2^ | 1.45* | 1.43* | 1.01 | 0.36 (6.3×10^-3^) |
| Niacinamide | 122.0480 | 1.25 | 1 | 4.4×10^-2^ | 1.07* | 1.05 | 1.02 | 0.40 (2.1×10^-3^) |
| *Krebs intermediates* | | | | | | | | |
| Succinic acid | 118.0266 | 1.40 | 1 | 7.0×10^-3^ | 1.60* | 1.48* | 1.08 | 0.37 (4.7×10^-3^) |
| *Carnitine-related metabolites* | | | | | | | | |
| 3-Hydroxy-trimethyllysine | 205.1552 | 0.61 | 2 | 3.8×10^-2^ | 1.17* | 1.21* | 0.97 |  |
| 3-Dehydroxycarnitine | 145.1103 | 0.70 | 1 | 3.9×10^-2^ | 1.08* | 1.15* | 0.94 | 0.28 (3.3×10^-2^) |
| Succinyl-L-carnitine | 261.1212 | 1.29 | 1 | 4.0×10^-2^ | 1.44* | 1.00 | 1.44* |  |
| Hydroxyhexanoyl-L-carnitine | 275.1733 | 2.22 | 3 | 4.0×10^-2^ | 1.15* | 1.16* | 0.99 | 0.25 (6.1×10^-2^) |
| *Free fatty acids* | | | | | | | | |
| Oleic acid | 282.2559 | 7.28 | 1 | 4.9×10^-2^ | 1.23* | 0.84 | 1.46* | 0.30 (2.5×10^-2^) |
| *Branched chain amino acids and derivatives* | | | | | | | | |
| L-Valine | 117.0790 | 0.88 | 1 | 4.0×10^-2^ | 1.42* | 1.31 | 1.08 | 0.42 (1.4×10^-3^) |
| L-Isoleucine | 131.0946 | 1.42 | 1 | 2.7×10^-2^ | 1.29* | 1.21* | 1.07 | 0.31 (1.8×10^-2^) |
| L-Leucine | 131.0946 | 1.48 | 1 | 4.8×10^-2^ | 1.51* | 1.35 | 1.11 | 0.36 (5.8×10^-3^) |
| Asparaginyl-leucine (or isomer) | 245.1376 | 1.34 | 3 | 4.4×10^-2^ | 1.55* | 1.09 | 1.43 | 0.44 (7.3×10^-4^) |
| Glutamyl-leucine (or isomer) | 260.1372 | 1.60 | 3 | 4.4×10^-2^ | 1.35* | 0.95 | 1.43 | 0.37 (4.8×10^-3^) |
| Leucyl-alanine | 202.1317 | 1.65 | 1 | 4.0×10^-2^ | 1.74* | 1.08 | 1.61* | 0.36 (5.7×10^-3^) |
| Leucyl-glycine | 188.1161 | 1.69 | 1 | 3.8×10^-2^ | 1.64* | 1.06 | 1.55* | 0.32 (1.8×10^-2^) |
| Valyl-valine | 216.1474 | 1.77 | 2 | 3.9×10^-2^ | 1.64* | 1.03 | 1.59* | 0.39 (2.9×10^-3^) |
| Seryl-leucine (isomer 1) | 218.1267 | 1.95 | 3 | 4.0×10^-2^ | 1.46* | 1.09 | 1.34* | 0.37 (4.9×10^-3^) |
| Seryl-leucine (isomer 2) | 218.1267 | 2.08 | 3 | 4.1×10^-2^ | 1.56* | 1.12 | 1.40 | 0.38 (3.7×10^-3^) |
| Threonyl-leucine (isomer 1) | 232.1423 | 2.08 | 3 | 4.0×10^-2^ | 2.19* | 1.10 | 1.99* | 0.28 (3.7×10^-2^) |
| Threonyl-leucine (isomer 2) | 232.1423 | 2.20 | 3 | 3.8×10^-2^ | 2.12* | 1.19 | 1.79* | 0.36 (6.5×10^-3^) |
| Valyl-isoleucine (or isomer) | 230.1630 | 2.26 | 3 | 4.8×10^-2^ | 1.60* | 1.05 | 1.52* | 0.34 (9.9×10^-3^) |
| Leucyl-valine | 230.1630 | 2.43 | 1 | 4.0×10^-2^ | 2.19* | 1.05 | 2.08* | 0.40 (2.3×10^-3^) |
| Valyl-leucine | 230.1630 | 2.63 | 1 | 4.9×10^-2^ | 1.44* | 1.07 | 1.34 | 0.48 (1.7×10^-4^) |
| Leucyl-leucine (or isomer) | 244.1787 | 3.20 | 3 | 4.0×10^-2^ | 2.20* | 1.14 | 1.93 | 0.36 (7.2×10^-3^) |
| Methionyl-leucine (or isomer) | 262.1351 | 4.21 | 3 | 4.8×10^-2^ | 1.15* | 1.08 | 1.07 | 0.42 (1.1×10^-3^) |
| 3-Aminoisobutyric acid | 103.0633 | 0.70 | 1 | 4.0×10^-2^ | 1.12* | 1.24* | 0.91 |  |
| 2-Hydroxyglutaric acid | 148.0372 | 1.11 | 1 | 7.0×10^-3^ | 1.37* | 1.01 | 1.36* | 0.38 (3.3×10^-3^) |
| 3-Methyl-2-oxobutyric acid | 116.0473 | 1.94 | 1 | 7.0×10^-3^ | 2.03* | 1.27 | 1.60* | 0.31 (1.9×10^-2^) |
| 3-Hydroxy-3-methylbutyric acid | 118.0630 | 2.08 | 1 | 4.7×10^-2^ | 1.67* | 1.47 | 1.14 |  |
| 2-Hydroxy-3-methylbutyric acid | 118.0630 | 2.67 | 1 | 4.4×10^-2^ | 1.80* | 1.27 | 1.42 | 0.31 (1.8×10^-2^) |
| 3-Methyl-2-oxovaleric acid | 130.0629 | 2.92 | 1 | 7.0×10^-3^ | 2.07* | 1.41 | 1.46 | 0.31 (1.8×10^-2^) |
| 4-Methyl-2-oxovaleric acid | 130.0629 | 3.08 | 1 | 7.0×10^-3^ | 2.09* | 1.32 | 1.59* | 0.33 (1.2×10^-2^) |
| *Aromatic amino acids and derivatives* | | | | | | | | |
| L-Tyrosine | 181.0738 | 1.29 | 1 | 4.0×10^-2^ | 1.35* | 1.24 | 1.09 | 0.43 (9.7×10^-4^) |
| L-Phenylalanine | 165.0790 | 1.99 | 1 | 4.1×10^-2^ | 1.45* | 1.30 | 1.12 | 0.31 (2.0×10^-2^) |
| L-Tryptophan | 204.0899 | 2.45 | 1 | 4.9×10^-2^ | 1.17* | 1.10 | 1.07 | 0.35 (7.7×10^-3^) |
| Aspartyl-phenylalanine (or isomer) | 280.1059 | 1.85 | 3 | 4.0×10^-2^ | 1.69* | 1.24 | 1.36 | 0.34 (9.4×10^-3^) |
| Seryl-phenylalanine (or isomer) | 252.1110 | 2.35 | 3 | 4.0×10^-2^ | 1.97* | 0.99 | 1.98* | 0.30 (2.4×10^-2^) |
| Threonyl-phenylalanine (or isomer) | 266.1267 | 2.50 | 3 | 1.7×10^-2^ | 2.25* | 1.21 | 1.86* | 0.40 (2.3×10^-3^) |
| Indoxyl sulfate | 213.0096 | 2.59 | 1 | 4.9×10^-2^ | 1.43* | 0.80 | 1.79* | 0.28 (3.6×10^-2^) |
| 4-Hydroxyphenyllactic acid | 182.0579 | 2.61 | 1 | 3.9×10^-3^ | 1.53* | 1.20 | 1.27* | 0.46 (2.9×10^-4^) |
| Dopamine | 153.0790 | 3.08 | 1 | 4.4×10^-2^ | 1.14* | 1.26* | 0.91 | 0.28 (3.5×10^-2^) |
| *Other amino acids* | | | | | | | | |
| L-Alanine | 89.0477 | 0.71 | 1 | 4.4×10^-2^ | 1.21* | 1.16 | 1.04 | 0.25 (6.3×10^-2^) |
| N-Acetylmethionine | 191.616 | 2.69 | 1 | 4.4×10^-2^ | 1.38* | 1.20 | 1.15 | 0.28 (3.6×10^-2^) |
| *Oxidative stress markers* | | | | | | | | |
| Cysteinyl-glycine | 178.0412 | 0.96 | 1 | 4.0×10^-2^ | 1.25* | 1.25 | 1.00 | 0.36 (6.4×10^-3^) |
| 2-Hydroxybutyric acid | 104.0473 | 1.72 | 1 | 3.5×10^-2^ | 1.72* | 1.33* | 1.29 | 0.31 (1.9×10^-2^) |
| 8-Hydroxyguanine | 167.0443 | 1.22 | 1 | 7.0×10^-3^ | 1.30* | 1.13 | 1.16* |  |
| 3-Nitrotyrosine | 226.0590 | 2.08 | 1 | 7.0×10^-3^ | 1.36* | 1.22* | 1.11 |  |
| Dityrosine | 360.1321 | 4.51 | 1 | 4.4×10^-2^ | 1.43* | 1.54* | 0.93 | 0.31 (1.8×10^-2^) |
| 4-Oxononenal glutathione | 461.1832 | 4.25 | 2 | 2.8×10^-2^ | 1.17* | 1.29* | 0.90 | 0.35 (7.4×10^-3^) |
| Leukoaminochrome | 151.0633 | 2.23 | 2 | 3.9×10^-2^ | 1.36* | 1.07 | 1.27 | 0.36 (2.8×10^-3^) |
| Adrenochrome / Adrenolutin | 179.0582 | 4.60 | 3 | 3.8×10^-2^ | 1.24* | 1.36* | 0.91 | 0.34 (1.1×10^-2^) |
| Adrenochrome / Adrenolutin | 179.0582 | 4.74 | 3 | 4.4×10^-2^ | 1.20* | 1.31* | 0.91 | 0.34 (1.0×10^-2^) |
| *Nucleotide metabolism* | | | | | | | | |
| Hypoxanthine | 136.0385 | 1.26 | 1 | 8.1×10^-3^ | 1.39* | 0.95 | 1.47* |  |
| Allantoin | 158.0440 | 0.72 | 1 | 4.5×10^-2^ | 2.65* | 1.98 | 1.34 | 0.26 (5.2×10^-2^) |
| 2’-Deoxyinosine | 252.0859 | 1.64 | 1 | 3.5×10^-2^ | 1.28* | 1.06 | 1.20* | 0.43 (7.8×10^-4^) |
| Ureidosuccinic acid | 176.0433 | 0.74 | 1 | 4.8×10^-2^ | 1.37* | 1.34 | 1.02 |  |
| *Fatty acid amides* | | | | | | | | |
| Linoleamide | 279.2562 | 6.87 | 2 | 4.0×10^-2^ | 0.81* | 0.96 | 0.84 |  |
| Palmitamide | 255.2562 | 6.96 | 2 | 3.9×10^-2^ | 0.62* | 0.90 | 0.69* |  |
| Stearamide | 283.2875 | 7.21 | 1 | 4.0×10^-2^ | 0.74* | 0.93 | 0.80 |  |
| *Phospholipids* | | | | | | | | |
| PC(14:0/18:2) | 729.5309 | 8.32 | 3 | 4.4×10^-2^ | 1.45* | 1.33* | 1.09 | 0.49 (1.2×10^-4^) |
| PC(16:0/16:1) | 731.5465 | 8.57 | 3 | 1.2×10^-2^ | 1.44* | 1.47* | 0.98 | 0.38 (3.5×10^-3^) |
| PC(16:0/20:4) | 781.5622 | 8.60 | 3 | 3.8×10^-2^ | 1.20* | 1.20* | 1.00 | 0.30 (2.5×10^-2^) |
| PE(18:1/18:2) | 741.5309 | 8.64 | 3 | 4.8×10^-2^ | 0.90* | 0.88* | 1.01 | -0.32 (1.4×10^-2^) |
| PE(P-16:0/20:4) | 723.5203 | 8.71 | 3 | 4.1×10^-2^ | 1.09* | 1.12* | 0.97 | 0.25 (6.2×10^-2^) |
| PE(P-18:1/20:4) | 749.5359 | 8.82 | 3 | 3.8×10^-2^ | 0.87* | 0.86* | 1.01 | -0.37 (5.3×10^-3^) |
| PC(16:0/20:3) | 783.5778 | 8.91 | 3 | 4.4×10^-2^ | 2.06* | 2.26* | 0.91 | 0.42 (1.3×10^-3^) |
| PE(18:1/18:1) | 743.5465 | 9.02 | 3 | 3.5×10^-2^ | 0.83* | 0.92 | 0.90 | -0.34 (1.1×10^-2^) |
| PE(P-18:0/18:1) | 729.5672 | 10.01 | 3 | 4.5×10^-2^ | 0.74* | 0.90 | 0.83 | -0.35 (7.6×10^-3^) |
| *Sphingolipids* | | | | | | | | |
| CER(d18:1/18:1) | 563.5277 | 8.66 | 2 | 4.8×10^-2^ | 1.25* | 1.17 | 1.07 | 0.28 (3.6×10^-2^) |
| CER(d18:1/22:0) | 621.6060 | 9.58 | 2 | 7.0×10^-3^ | 1.54* | 1.50* | 1.03 | 0.41 (1.4×10^-3^) |
| SM(d18:1/18:1) | 728.5832 | 8.56 | 2 | 4.9×10^-2^ | 1.19* | 1.16 | 1.02 | 0.25 (6.6×10^-2^) |
| SM(d16:1/22:0) | 758.6302 | 8.87 | 2 | 3.8×10^-2^ | 1.14* | 1.20* | 0.95 |  |
| *Diet-related metabolites* | | | | | | | | |
| Proline betaine | 143.0946 | 0.74 | 1 | 4.1×10^-2^ | 0.65* | 0.87 | 0.75 | -0.42 (1.2×10^-3^) |
| 4-Hydroxybenzaldehyde | 122.0368 | 3.22 | 1 | 4.4×10^-2^ | 0.86* | 1.02 | 0.85* |  |
| Caffeine | 194.0804 | 3.06 | 1 | 4.0×10^-2^ | 1.39* | 1.08 | 1.29 |  |
| *Exposome-related metabolites* | | | | | | | | |
| 1-Hydroxy-2-naphthoic acid | 188.0473 | 0.94 | 2 | 4.5×10^-2^ | 1.61* | 0.77 | 2.08* |  |

**Table 3.** Differential plasma metabolites along the oral glucose tolerance test in children with obesity and insulin resistance (ObIR+) and children with obesity without insulin resistance (ObIR-). * Indicates significant differences according to the post-hoc Fisher LSD test (p < 0.05).

| **Metabolite** | **Mass (Da)** | **RT (min)** | **MSI level** | **group** | **p value** | **Fold changes** | | |
| --- | --- | --- | --- | --- | --- | --- | --- | --- |
|  |  |  |  |  |  | **60 vs 0** | **120 vs 0** | **120 vs 60** |
| *Glycolytic intermediates and related metabolites* | | | | | | | | |
| D-Glucose (or isomer) | 180.0634 | 0.71 | 1 | ObIR- | 1.2×10^-3^ | 1.74* | 1.19 | 0.74* |
|  |  |  |  | ObIR+ | 4.4×10^-10^ | 2.10* | 2.09* | 0.99 |
| D-Glucose 6-sulfate (or isomer) | 260.0202 | 0.71 | 3 | ObIR- | 9.5×10^-3^ | 1.25* | 1.11 | 0.89* |
|  |  |  |  | ObIR+ | 2.2×10^-9^ | 1.33* | 1.37* | 1.03 |
| D-Gluconic acid (or isomer) | 196.0583 | 0.71 | 1 | ObIR- | 2.2×10^-2^ | 1.29* | 1.00 | 0.78* |
|  |  |  |  | ObIR+ | 1.7×10^-5^ | 1.42* | 1.38* | 0.97 |
| 1,6-Anhydroglucose (or isomer) | 162.0528 | 0.76 | 1 | ObIR- | 2.0×10^-1^ | 1.42 | 1.45 | 1.02 |
|  |  |  |  | ObIR+ | 1.8×10^-3^ | 1.36* | 1.46* | 1.07 |
| Lactic acid | 90.0317 | 0.89 | 1 | ObIR- | 4.5×10^-2^ | 1.37* | 1.07 | 0.78* |
|  |  |  |  | ObIR+ | 2.6×10^-3^ | 1.40* | 1.21 | 0.86 |
| Creatinine | 113.0589 | 0.68 | 1 | ObIR- | 3.6×10^-2^ | 1.17* | 1.03 | 0.88* |
|  |  |  |  | ObIR+ | 3.8×10^-2^ | 1.10 | 1.14* | 1.04 |
| *Ketone bodies* | | | | | | | | |
| Acetoacetic acid | 102.0317 | 0.89 | 1 | ObIR- | 1.4×10^-2^ | 0.86 | 0.60* | 0.70* |
|  |  |  |  | ObIR+ | 5.1×10^-1^ | 1.01 | 0.97 | 0.96 |
| 3-Hydroxybutyric acid | 104.0473 | 1.46 | 1 | ObIR- | 7.2×10^-5^ | 0.26* | 0.10* | 0.37 |
|  |  |  |  | ObIR+ | 2.7×10^-7^ | 0.27* | 0.28* | 1.03 |
| *Carnitine-related metabolites* | | | | | | | | |
| Undecenoyl-L-carnitine | 327.2409 | 5.17 | 3 | ObIR- | 4.9×10^-2^ | 0.88* | 0.78* | 0.89 |
|  |  |  |  | ObIR+ | 2.0×10^-2^ | 0.90* | 0.88* | 0.97 |
| Hydroxytetradecadienoyl-L-carnitine | 383.2672 | 5.38 | 3 | ObIR- | 2.0×10^-3^ | 0.60* | 0.56* | 0.93 |
|  |  |  |  | ObIR+ | 5.9×10^-4^ | 0.70* | 0.68* | 0.97 |
| Myristoleoyl-L-carnitine | 369.2879 | 5.85 | 2 | ObIR- | 4.9×10^-2^ | 0.88 | 0.81* | 0.92 |
|  |  |  |  | ObIR+ | 1.1×10^-2^ | 0.83* | 0.82* | 0.99 |
| Oleoyl-L-carnitine | 425.3505 | 6.32 | 1 | ObIR- | 4.9×10^-2^ | 0.87 | 0.78* | 0.90 |
|  |  |  |  | ObIR+ | 5.5×10^-4^ | 0.84* | 0.75* | 0.90 |
| *Hydroxylated fatty acids* | | | | | | | | |
| Hydroxyhexanoic acid | 132.0786 | 3.23 | 3 | ObIR- | 2.0×10^-2^ | 0.70 | 0.51* | 0.73 |
|  |  |  |  | ObIR+ | 7.1×10^-2^ | 0.73 | 0.71 | 0.97 |
| 2-Hydroxyoctanoic acid | 160.1099 | 5.02 | 1 | ObIR- | 4.5×10^-2^ | 0.82 | 0.67* | 0.82 |
|  |  |  |  | ObIR+ | 6.7×10^-1^ | 0.95 | 0.91 | 0.96 |
| Hydroxyoctanoic acid isomer | 160.1099 | 4.72 | 3 | ObIR- | 3.6×10^-4^ | 0.59* | 0.47* | 0.79 |
|  |  |  |  | ObIR+ | 6.1×10^-8^ | 0.58* | 0.55* | 0.95 |
| Hydroxyoctenoic acid | 158.943 | 4.79 | 3 | ObIR- | 1.7×10^-2^ | 0.69 | 0.51* | 0.74 |
|  |  |  |  | ObIR+ | 4.9×10^-2^ | 0.84 | 0.74* | 0.89 |
| Hydroxynonanoic acid | 174.1256 | 5.21 | 3 | ObIR- | 2.7×10^-2^ | 0.93 | 0.80* | 0.86 |
|  |  |  |  | ObIR+ | 1.3×10^-3^ | 0.85* | 0.86* | 1.01 |
| Hydroxydecanoic acid (isomer 1) | 188.1412 | 4.64 | 3 | ObIR- | 1.8×10^-4^ | 0.51* | 0.43* | 0.85 |
|  |  |  |  | ObIR+ | 1.2×10^-6^ | 0.54* | 0.45* | 0.84 |
| Hydroxydecanoic acid (isomer 2) | 188.1412 | 5.60 | 3 | ObIR- | 1.1×10^-7^ | 0.36* | 0.27* | 0.75 |
|  |  |  |  | ObIR+ | 1.7×10^-14^ | 0.40* | 0.35* | 0.86 |
| Hydroxydecanoic acid (isomer 3) | 188.1412 | 5.81 | 3 | ObIR- | 7.1×10^-6^ | 0.65* | 0.53* | 0.81 |
|  |  |  |  | ObIR+ | 2.0×10^-6^ | 0.69* | 0.59* | 0.85 |
| Hydroxydecenoic acid (isomer 1) | 186.1256 | 5.54 | 3 | ObIR- | 9.0×10^-4^ | 0.57* | 0.39* | 0.68 |
|  |  |  |  | ObIR+ | 4.5×10^-6^ | 0.62* | 0.45* | 0.72 |
| Hydroxydecenoic acid (isomer 2) | 186.1256 | 5.75 | 3 | ObIR- | 3.0×10^-2^ | 0.75* | 0.68* | 0.91 |
|  |  |  |  | ObIR+ | 5.9×10^-5^ | 0.74* | 0.66* | 0.90 |
| Hydroxydecadienoic acid (isomer 1) | 184.1099 | 5.19 | 3 | ObIR- | 8.3×10^-2^ | 0.75 | 0.65 | 0.87 |
|  |  |  |  | ObIR+ | 9.9×10^-3^ | 0.87 | 0.71* | 0.82 |
| Hydroxydecadienoic acid (isomer 2) | 184.1099 | 5.32 | 3 | ObIR- | 3.1×10^-2^ | 0.70* | 0.66* | 0.95 |
|  |  |  |  | ObIR+ | 5.1×10^-1^ | 0.95 | 0.88 | 0.92 |
| Hydroxyundecanoic acid | 202.1569 | 5.83 | 3 | ObIR- | 7.3×10^-3^ | 0.73 | 0.46* | 0.62 |
|  |  |  |  | ObIR+ | 1.9×10^-4^ | 0.73* | 0.68* | 0.93 |
| 3-Hydroxylauric acid | 216.1725 | 6.16 | 1 | ObIR- | 1.1×10^-8^ | 0.35* | 0.31* | 0.89* |
|  |  |  |  | ObIR+ | 3.4×10^-16^ | 0.40* | 0.37* | 0.92 |
| Hydroxylauric acid isomer | 216.1725 | 5.98 | 3 | ObIR- | 9.3×10^-3^ | 0.83* | 0.78* | 0.95 |
|  |  |  |  | ObIR+ | 2.8×10^-4^ | 0.79* | 0.74* | 0.93 |
| Hydroxydodecenoic acid | 214.1569 | 5.94 | 3 | ObIR- | 1.3×10^-8^ | 0.37* | 0.31* | 0.83 |
|  |  |  |  | ObIR+ | 7.6×10^-17^ | 0.39* | 0.35* | 0.91 |
| Hydroxydodecadienoic acid | 212.1412 | 5.65 | 3 | ObIR- | 4.9×10^-1^ | 1.01 | 0.98 | 0.97 |
|  |  |  |  | ObIR+ | 2.5×10^-2^ | 0.76* | 0.74* | 0.97 |
| 3-Hydroxymyristic acid | 244.2038 | 6.55 | 1 | ObIR- | 9.8×10^-9^ | 0.32* | 0.29* | 0.89 |
|  |  |  |  | ObIR+ | 1.4×10^-17^ | 0.37* | 0.31* | 0.85 |
| Hydroxytetradecenoic acid | 242.1882 | 6.41 | 3 | ObIR- | 1.7×10^-8^ | 0.28* | 0.21* | 0.76 |
|  |  |  |  | ObIR+ | 1.3×10^-17^ | 0.30* | 0.26* | 0.85* |
| Hydroxytetradecadienoic acid (isomer 1) | 240.1725 | 6.20 | 3 | ObIR- | 3.6×10^-8^ | 0.28* | 0.22* | 0.77 |
|  |  |  |  | ObIR+ | 2.2×10^-15^ | 0.31* | 0.26* | 0.83 |
| Hydroxytetradecadienoic acid (isomer 2) | 240.1725 | 6.66 | 3 | ObIR- | 5.5×10^-3^ | 0.54* | 0.40* | 0.75 |
|  |  |  |  | ObIR+ | 3.8×10^-4^ | 0.66* | 0.46* | 0.71 |
| 16-Hydroxypalmitic acid | 272.2351 | 6.47 | 1 | ObIR- | 9.8×10^-9^ | 0.48* | 0.49* | 1.02 |
|  |  |  |  | ObIR+ | 1.6×10^-13^ | 0.39* | 0.37* | 0.95 |
| 2-Hydroxypalmitic acid | 272.2351 | 6.94 | 1 | ObIR- | 2.4×10^-5^ | 0.70* | 0.62* | 0.90 |
|  |  |  |  | ObIR+ | 1.7×10^-12^ | 0.74* | 0.62* | 0.84* |
| Hydroxypalmitic acid isomer | 272.2351 | 6.84 | 3 | ObIR- | 5.0×10^-7^ | 0.56* | 0.58* | 1.04 |
|  |  |  |  | ObIR+ | 2.2×10^-11^ | 0.48* | 0.46* | 0.95 |
| Hydroxyhexadecadienoic acid | 268.2038 | 6.50 | 3 | ObIR- | 3.7×10^-7^ | 0.35* | 0.34* | 0.96 |
|  |  |  |  | ObIR+ | 2.3×10^-14^ | 0.32* | 0.34* | 1.04 |
| 12-Hydroxystearic acid | 300.2664 | 6.75 | 1 | ObIR- | 9.8×10^-9^ | 0.46* | 0.41* | 0.90 |
|  |  |  |  | ObIR+ | 2.2×10^-11^ | 0.33* | 0.31* | 0.94 |
| 2-Hydroxystearic acid | 300.2664 | 7.18 | 1 | ObIR- | 3.6×10^-4^ | 0.75* | 0.65* | 0.87 |
|  |  |  |  | ObIR+ | 1.0×10^-6^ | 0.76* | 0.67* | 0.88 |
| Hydroxystearic acid isomer | 300.2664 | 7.07 | 3 | ObIR- | 3.2×10^-5^ | 0.68* | 0.60* | 0.89 |
|  |  |  |  | ObIR+ | 2.3×10^-9^ | 0.52* | 0.44* | 0.84 |
| Dihydroxystearic acid | 316.2614 | 6.38 | 3 | ObIR- | 3.0×10^-1^ | 0.79 | 0.84 | 1.06 |
|  |  |  |  | ObIR+ | 1.4×10^-2^ | 0.76* | 0.73* | 0.96 |
| 12-Hydroxyoleic acid | 298.2508 | 6.61 | 1 | ObIR- | 3.8×10^-8^ | 0.48* | 0.35* | 0.73 |
|  |  |  |  | ObIR+ | 1.1×10^-15^ | 0.31* | 0.31* | 0.98 |
| Hydroxyoleic acid isomer | 298.2508 | 6.91 | 3 | ObIR- | 8.1×10^-7^ | 0.31* | 0.23* | 0.74 |
|  |  |  |  | ObIR+ | 8.8×10^-16^ | 0.33* | 0.26* | 0.79* |
| Dihydroxyoctadecenoic acid (DiHOME) | 314.2457 | 6.21 | 3 | ObIR- | 3.8×10^-5^ | 0.66* | 0.39* | 0.59 |
|  |  |  |  | ObIR+ | 7.4×10^-11^ | 0.51* | 0.36* | 0.69* |
| Hydroxyoctadecadienoic acid (HODE) (isomer 1) | 296.2351 | 6.49 | 3 | ObIR- | 5.4×10^-4^ | 0.21* | 0.38* | 1.80 |
|  |  |  |  | ObIR+ | 6.7×10^-6^ | 0.34* | 0.54* | 1.59 |
| Hydroxyoctadecadienoic acid (HODE) (isomer 2) | 296.2351 | 6.76 | 3 | ObIR- | 1.1×10^-8^ | 0.32* | 0.23* | 0.74 |
|  |  |  |  | ObIR+ | 4.3×10^-16^ | 0.33* | 0.25* | 0.75* |
| Dihydroxyoctadecadienoic acid (DiHODE) | 312.2301 | 6.17 | 3 | ObIR- | 4.8×10^-4^ | 0.42* | 0.66* | 1.58 |
|  |  |  |  | ObIR+ | 9.1×10^-2^ | 0.58 | 0.53 | 0.92 |
| Hydroxyoctadecatrienoic acid (HOTrE) | 294.2195 | 6.66 | 3 | ObIR- | 9.8×10^-9^ | 0.48* | 0.41* | 0.85 |
|  |  |  |  | ObIR+ | 1.3×10^-17^ | 0.46* | 0.38* | 0.83* |
| *Free fatty acids* | | | | | | | | |
| Dodecadienoic acid | 196.1463 | 6.34 | 3 | ObIR- | 2.1×10^-6^ | 0.34* | 0.29* | 0.85 |
|  |  |  |  | ObIR+ | 1.1×10^-16^ | 0.32* | 0.26* | 0.82 |
| Lauric acid | 200.1776 | 6.67 | 1 | ObIR- | 7.0×10^-1^ | 1.16 | 0.99 | 0.85 |
|  |  |  |  | ObIR+ | 2.1×10^-3^ | 0.49* | 0.60* | 1.03 |
| Hexadecatrienoic acid | 250.1933 | 6.78 | 3 | ObIR- | 1.4×10^-8^ | 0.30* | 0.22* | 0.75 |
|  |  |  |  | ObIR+ | 4.3×10^-16^ | 0.27* | 0.21* | 0.79* |
| Myristoleic acid | 226.1933 | 6.79 | 1 | ObIR- | 5.7×10^-5^ | 0.22* | 0.16* | 0.72 |
|  |  |  |  | ObIR+ | 1.1×10^-9^ | 0.18* | 0.17* | 0.94 |
| (5Z,8Z,11Z,14Z,17Z)-Eicosapentaenoic acid | 302.2246 | 6.95 | 1 | ObIR- | 3.2×10^-5^ | 0.29* | 0.28* | 0.95 |
|  |  |  |  | ObIR+ | 2.5×10^-6^ | 0.47* | 0.38* | 0.80 |
| Myristic acid | 228.2089 | 6.95 | 1 | ObIR- | 8.7×10^-6^ | 0.45* | 0.34* | 0.76 |
|  |  |  |  | ObIR+ | 1.2×10^-10^ | 0.39* | 0.41* | 1.04 |
| Linolenic acid | 278.2246 | 6.97 | 1 | ObIR- | 1.1×10^-8^ | 0.28* | 0.19* | 0.74 |
|  |  |  |  | ObIR+ | 3.3×10^-16^ | 0.28* | 0.22* | 0.77* |
| Palmitoleic acid | 254.2246 | 7.02 | 1 | ObIR- | 5.5×10^-5^ | 0.34* | 0.21* | 0.62 |
|  |  |  |  | ObIR+ | 2.3×10^-12^ | 0.17* | 0.28* | 1.65 |
| (4Z,7Z,10Z,13Z,16Z,19Z)-Docosahexaenoic acid | 328.2402 | 7.05 | 1 | ObIR- | 1.4×10^-5^ | 0.44* | 0.31* | 0.72 |
|  |  |  |  | ObIR+ | 1.1×10^-8^ | 0.55* | 0.37* | 0.68* |
| Arachidonic acid | 304.2402 | 7.08 | 1 | ObIR- | 1.6×10^-7^ | 0.42* | 0.43* | 1.02 |
|  |  |  |  | ObIR+ | 2.8×10^-10^ | 0.43* | 0.43* | 1.00 |
| Linoleic acid | 280.2402 | 7.10 | 1 | ObIR- | 8.1×10^-6^ | 0.29* | 0.18* | 0.61 |
|  |  |  |  | ObIR+ | 7.0×10^-10^ | 0.30* | 0.20* | 0.69 |
| (4Z,7Z,10Z,13Z,16Z)-Docosapentaenoic acid | 330.2559 | 7.18 | 1 | ObIR- | 1.6×10^-7^ | 0.38* | 0.28* | 0.73 |
|  |  |  |  | ObIR+ | 3.2×10^-11^ | 0.42* | 0.32* | 0.76 |
| (8Z,11Z,14Z)-Eicosatrienoic acid | 306.2559 | 7.20 | 1 | ObIR- | 1.7×10^-8^ | 0.36* | 0.31* | 0.86 |
|  |  |  |  | ObIR+ | 6.0×10^-13^ | 0.38* | 0.31* | 0.83 |
| Palmitic acid | 256.2402 | 7.22 | 1 | ObIR- | 1.4×10^-8^ | 0.38* | 0.26* | 0.68 |
|  |  |  |  | ObIR+ | 6.3×10^-17^ | 0.40* | 0.35* | 0.88 |
| Oleic acid | 282.2559 | 7.28 | 1 | ObIR- | 1.4×10^-8^ | 0.29* | 0.17* | 0.60 |
|  |  |  |  | ObIR+ | 1.5×10^-17^ | 0.28* | 0.21* | 0.76* |
| Docosatetraenoic acid | 332.2715 | 7.29 | 3 | ObIR- | 1.0×10^-7^ | 0.35* | 0.29* | 0.84 |
|  |  |  |  | ObIR+ | 5.3×10^-13^ | 0.34* | 0.29* | 0.85 |
| Margaric acid | 270.2559 | 7.35 | 1 | ObIR- | 4.8×10^-3^ | 0.64* | 0.52* | 0.82 |
|  |  |  |  | ObIR+ | 6.2×10^-4^ | 0.45* | 0.56* | 1.24 |
| Eicosadienoic acid | 308.2715 | 7.37 | 3 | ObIR- | 1.1×10^-7^ | 0.38* | 0.24* | 0.61 |
|  |  |  |  | ObIR+ | 1.5×10^-15^ | 0.35* | 0.26* | 0.76* |
| Tetracosapentaenoic acid | 358.2872 | 7.39 | 3 | ObIR- | 2.8×10^-1^ | 0.87 | 0.72 | 0.83 |
|  |  |  |  | ObIR+ | 4.9×10^-4^ | 0.37* | 0.30* | 0.87 |
| Eicosenoic acid | 310.2872 | 7.59 | 3 | ObIR- | 8.8×10^-5^ | 0.56* | 0.26* | 0.46 |
|  |  |  |  | ObIR+ | 1.1×10^-10^ | 0.21* | 0.13* | 0.64* |
| *Branched chain amino acids and derivatives* | | | | | | | | |
| L-Valine | 117.0790 | 0.87 | 1 | ObIR- | 8.9×10^-3^ | 0.85 | 0.73* | 0.86 |
|  |  |  |  | ObIR+ | 3.8×10^-2^ | 0.88 | 0.82* | 0.93 |
| L-Leucine | 131.0946 | 1.50 | 1 | ObIR- | 9.4×10^-4^ | 0.72* | 0.60* | 0.83 |
|  |  |  |  | ObIR+ | 2.6×10^-4^ | 0.75* | 0.69* | 0.92 |
| 3-Aminoisobutyric acid | 103.0633 | 0.71 | 1 | ObIR- | 4.9×10^-2^ | 0.73 | 0.69* | 0.95 |
|  |  |  |  | ObIR+ | 2.1×10^-1^ | 0.95 | 0.89 | 0.94 |
| 3-Methyl-2-oxobutyric acid | 116.0473 | 1.84 | 1 | ObIR- | 4.7×10^-2^ | 0.83 | 0.64* | 0.78 |
|  |  |  |  | ObIR+ | 8.1×10^-1^ | 0.98 | 1.00 | 1.03 |
| 3-Methyl-2-oxovaleric acid | 130.0629 | 2.77 | 1 | ObIR- | 8.5×10^-5^ | 0.62* | 0.42* | 0.68* |
|  |  |  |  | ObIR+ | 1.4×10^-10^ | 0.67* | 0.43* | 0.65* |
| 4-Methyl-2-oxovaleric acid | 130.0629 | 2.93 | 1 | ObIR- | 2.9×10^-4^ | 0.46* | 0.33* | 0.72 |
|  |  |  |  | ObIR+ | 7.0×10^-10^ | 0.67* | 0.38* | 0.57* |
| *Aromatic amino acids and derivatives* | | | | | | | | |
| L-Tyrosine | 181.0738 | 1.34 | 1 | ObIR- | 2.3×10^-5^ | 0.73* | 0.63* | 0.86 |
|  |  |  |  | ObIR+ | 2.2×10^-11^ | 0.72* | 0.64* | 0.89 |
| L-Phenylalanine | 165.0790 | 2.01 | 1 | ObIR- | 1.1×10^-2^ | 0.81* | 0.69* | 0.85 |
|  |  |  |  | ObIR+ | 2.9×10^-2^ | 0.68* | 0.68* | 1.01 |
| L-Tryptophan | 204.0899 | 2.45 | 1 | ObIR- | 4.9×10^-2^ | 0.88 | 0.69* | 0.79 |
|  |  |  |  | ObIR+ | 8.3×10^-1^ | 0.92 | 0.95 | 1.04 |
| Hydroxyphenylacetic acid sulfate | 232.0042 | 2.50 | 3 | ObIR- | 2.1×10^-2^ | 0.66* | 0.56* | 0.85 |
|  |  |  |  | ObIR+ | 6.7×10^-3^ | 0.61* | 0.48* | 0.78 |
| 4-Hydroxyphenylpyruvic acid | 180.0423 | 2.06 | 1 | ObIR- | 2.0×10^-2^ | 0.94 | 0.68* | 0.73* |
|  |  |  |  | ObIR+ | 4.5×10^-1^ | 0.94 | 0.91 | 0.97 |
| p-Cresol | 108.0575 | 4.18 | 1 | ObIR- | 2.6×10^-1^ | 1.03 | 1.02 | 0.99 |
|  |  |  |  | ObIR+ | 1.1×10^-2^ | 0.96* | 0.97* | 1.00 |
| *Other amino acids* | | | | | | | | |
| L-Glutamine | 146.0691 | 0.66 | 1 | ObIR- | 1.6×10^-2^ | 0.87* | 0.84* | 0.97 |
|  |  |  |  | ObIR+ | 2.0×10^-2^ | 0.94 | 0.89* | 0.95 |
| L-Threonine | 119.0582 | 0.70 | 1 | ObIR- | 2.4×10^-2^ | 0.81 | 0.68* | 0.84 |
|  |  |  |  | ObIR+ | 7.7×10^-4^ | 0.73* | 0.69* | 0.95 |
| L-Proline | 115.0633 | 0.74 | 1 | ObIR- | 7.4×10^-2^ | 0.96 | 0.91 | 0.95 |
|  |  |  |  | ObIR+ | 1.8×10^-5^ | 0.86* | 0.89* | 1.03 |
| L-Methionine | 149.0510 | 0.89 | 1 | ObIR- | 1.1×10^-6^ | 0.74* | 0.60* | 0.81* |
|  |  |  |  | ObIR+ | 9.9×10^-11^ | 0.72* | 0.65* | 0.89 |
| Glutamyl-aspartic acid (or isomer) | 262.0801 | 0.87 | 3 | ObIR- | 2.4×10^-2^ | 0.93 | 0.83* | 0.89 |
|  |  |  |  | ObIR+ | 8.3×10^-1^ | 0.96 | 0.99 | 1.03 |
| γ-Glutamyl-leucine | 260.1372 | 2.84 | 1 | ObIR- | 3.9×10^-1^ | 0.73 | 0.98 | 1.34 |
|  |  |  |  | ObIR+ | 1.3×10^-2^ | 0.49* | 0.42* | 0.86 |
| cyclo(Prolyl-valine) | 196.1212 | 3.04 | 1 | ObIR- | 3.2×10^-3^ | 0.86 | 0.49* | 0.57* |
|  |  |  |  | ObIR+ | 1.0×10^-2^ | 0.83 | 0.67* | 0.81* |
| *Nucleotide metabolism* | | | | | | | | |
| Xanthine | 152.0334 | 1.31 | 1 | ObIR- | 4.1×10^-2^ | 0.87 | 0.72* | 0.83 |
|  |  |  |  | ObIR+ | 1.6×10^-3^ | 0.83* | 0.74* | 0.90 |
| *Corticosteroids* | | | | | | | | |
| Cortisone | 360.1937 | 4.84 | 1 | ObIR- | 4.7×10^-2^ | 0.73* | 0.82 | 1.12 |
|  |  |  |  | ObIR+ | 9.8×10^-7^ | 0.76* | 0.64* | 0.85 |
| Cortisol | 362.2093 | 5.00 | 1 | ObIR- | 9.8×10^-4^ | 0.51* | 0.58* | 1.13 |
|  |  |  |  | ObIR+ | 2.5×10^-6^ | 0.64* | 0.49* | 0.77 |
| Tetrahydrocortisol | 366.2406 | 5.40 | 1 | ObIR- | 4.0×10^-2^ | 0.78 | 0.70 | 0.89 |
|  |  |  |  | ObIR+ | 6.7×10^-6^ | 0.77* | 0.64* | 0.83 |
| *Bile acids* | | | | | | | | |
| Cholic acid | 408.2876 | 6.30 | 1 | ObIR- | 2.7×10^-2^ | 0.69 | 0.23* | 0.33 |
|  |  |  |  | ObIR+ | 7.2×10^-3^ | 0.58* | 0.43* | 0.75 |
| Deoxycholic acid | 392.2927 | 6.60 | 1 | ObIR- | 2.4×10^-2^ | 0.44 | 0.25* | 0.57 |
|  |  |  |  | ObIR+ | 4.7×10^-4^ | 0.54* | 0.40* | 0.74 |
| 12α-Hydroxy-3-oxocholadienic acid | 386.2457 | 5.82 | 2 | ObIR- | 4.9×10^-2^ | 0.99 | 0.88* | 0.89 |
|  |  |  |  | ObIR+ | 4.6×10^-1^ | 0.96 | 0.92 | 0.96 |
| Hydroxyoxocholestenoic acid | 430.3083 | 6.64 | 3 | ObIR- | 2.7×10^-1^ | 0.99 | 1.02 | 1.04 |
|  |  |  |  | ObIR+ | 1.8×10^-2^ | 0.81* | 0.85* | 1.05 |
| Hydroxycholestenone | 400.3341 | 7.14 | 3 | ObIR- | 4.9×10^-2^ | 0.77 | 0.69* | 0.89 |
|  |  |  |  | ObIR+ | 4.2×10^-2^ | 0.69* | 0.78 | 1.14 |
| *Phospholipids* | | | | | | | | |
| LPE(22:6) | 525.2855 | 6.78 | 3 | ObIR- | 4.1×10^-2^ | 0.86 | 0.82* | 0.96 |
|  |  |  |  | ObIR+ | 6.9×10^-1^ | 1.06 | 0.97 | 0.92 |
| LPE(18:1) | 479.3012 | 6.96 | 3 | ObIR- | 2.4×10^-2^ | 0.83 | 0.77* | 0.92 |
|  |  |  |  | ObIR+ | 1.1×10^-2^ | 0.95 | 0.80* | 0.84* |
| LPE(P-18:1) | 463.3063 | 7.09 | 3 | ObIR- | 4.1×10^-2^ | 0.39* | 0.57* | 1.44 |
|  |  |  |  | ObIR+ | 2.6×10^-4^ | 0.54* | 0.61* | 1.13 |
| *Other metabolites* | | | | | | | | |
| Hippuric acid | 179.0582 | 2.90 | 1 | ObIR- | 3.2×10^-5^ | 2.55* | 2.29* | 0.90 |
|  |  |  |  | ObIR+ | 1.3×10^-7^ | 2.06* | 2.08* | 1.01 |
| 4-Methylhippuric acid | 193.0739 | 3.44 | 1 | ObIR- | 4.2×10^-2^ | 2.04* | 1.57 | 0.77 |
|  |  |  |  | ObIR+ | 5.8×10^-4^ | 1.69* | 1.67* | 0.99 |

**Table 4.** Differential erythroid metabolites along the oral glucose tolerance test in children with obesity and insulin resistance (ObIR+) and children with obesity without insulin resistance (ObIR-). * Indicates significant differences according to the post-hoc Fisher LSD test (p < 0.05).

| **Metabolite** | **Mass (Da)** | **RT (min)** | **MSI level** | **group** | **p value** | **Fold changes** | | |
| --- | --- | --- | --- | --- | --- | --- | --- | --- |
|  |  |  |  |  |  | **60 vs 0** | **120 vs 0** | **120 vs 60** |
| *Glycolytic intermediates and related metabolites* | | | | | | | | |
| D-Ribose 5-phosphate (or isomer) | 230.0192 | 0.68 | 1 | ObIR- | 4.9×10^-2^ | 1.06 | 1.19* | 1.12 |
|  |  |  |  | ObIR+ | 3.9×10^-2^ | 1.21* | 1.18* | 0.98 |
| *Krebs intermediates* | | | | | | | | |
| Succinic acid | 118.0266 | 1.40 | 1 | ObIR- | 4.9×10^-2^ | 0.83 | 0.77* | 0.92 |
|  |  |  |  | ObIR+ | 1.5×10^-2^ | 0.81* | 0.75* | 0.93 |
| *Free fatty acids* | | | | | | | | |
| Palmitic acid | 256.2402 | 7.22 | 1 | ObIR- | 4.9×10^-2^ | 0.83 | 0.77* | 0.94 |
|  |  |  |  | ObIR+ | 3.4×10^-2^ | 0.86* | 0.84* | 0.98 |
| Oleic acid | 282.2559 | 7.28 | 1 | ObIR- | 4.9×10^-2^ | 0.78 | 0.65* | 0.83 |
|  |  |  |  | ObIR+ | 3.3×10^-2^ | 0.91 | 0.66* | 0.72 |
| Stearic acid | 284.2715 | 7.54 | 1 | ObIR- | 1.3×10^-3^ | 0.77* | 0.74* | 0.96 |
|  |  |  |  | ObIR+ | 4.9×10^-4^ | 0.82* | 0.79* | 0.96 |
| *Branched chain amino acids and derivatives* | | | | | | | | |
| L-Leucine | 131.0946 | 1.48 | 1 | ObIR- | 4.9×10^-2^ | 0.71* | 0.71* | 0.99 |
|  |  |  |  | ObIR+ | 3.8×10^-3^ | 0.86* | 0.77* | 0.90 |
| 3-Methyl-2-oxovaleric acid | 130.0629 | 2.92 | 1 | ObIR- | 1.8×10^-2^ | 0.79 | 0.61* | 0.78 |
|  |  |  |  | ObIR+ | 9.3×10^-4^ | 0.67* | 0.60* | 0.90 |
| 4-Methyl-2-oxovaleric acid | 130.0629 | 3.09 | 1 | ObIR- | 4.9×10^-2^ | 0.84 | 0.67* | 0.80 |
|  |  |  |  | ObIR+ | 2.2×10^-3^ | 0.71* | 0.63* | 0.89 |
| *Aromatic amino acids and derivatives* | | | | | | | | |
| L-Tyrosine | 181.0738 | 1.30 | 1 | ObIR- | 8.8×10^-3^ | 0.75* | 0.73* | 0.97 |
|  |  |  |  | ObIR+ | 3.3×10^-5^ | 0.80* | 0.70* | 0.88 |
| L-Tryptophan | 204.0899 | 2.44 | 1 | ObIR- | 3.2×10^-2^ | 0.72* | 0.70* | 0.97 |
|  |  |  |  | ObIR+ | 3.1×10^-5^ | 0.78* | 0.70* | 0.90 |
| 5-Hydroxytryptophan | 220.0848 | 1.62 | 1 | ObIR- | 3.3×10^-5^ | 0.83* | 0.80* | 0.96 |
|  |  |  |  | ObIR+ | 5.6×10^-5^ | 0.84* | 0.83* | 0.99 |
| Kynurenine | 208.0848 | 1.80 | 1 | ObIR- | 4.5×10^-2^ | 0.86* | 0.77* | 0.89 |
|  |  |  |  | ObIR+ | 8.2×10^-3^ | 0.86* | 0.86* | 0.99 |
| 4-Hydroxyphenyllactic acid | 182.0579 | 2.61 | 1 | ObIR- | 2.4×10^-2^ | 0.88 | 0.76* | 0.86 |
|  |  |  |  | ObIR+ | 1.9×10^-2^ | 0.87 | 0.80* | 0.91 |
| *Other amino acids* | | | | | | | | |
| L-Lysine | 146.1055 | 0.58 | 1 | ObIR- | 4.9×10^-2^ | 0.77* | 0.78* | 1.01 |
|  |  |  |  | ObIR+ | 9.0×10^-3^ | 0.87* | 0.79* | 0.90 |
| L-Threonine | 119.0582 | 0.70 | 1 | ObIR- | 2.0×10^-2^ | 0.83 | 0.68* | 0.82 |
|  |  |  |  | ObIR+ | 7.1×10^-4^ | 0.81 | 0.72* | 0.89 |
| L-Proline | 115.0633 | 0.74 | 1 | ObIR- | 1.0×10^-2^ | 0.76* | 0.73* | 0.96 |
|  |  |  |  | ObIR+ | 4.1×10^-4^ | 0.85* | 0.82* | 0.96 |
| L-Methionine | 149.0510 | 0.96 | 1 | ObIR- | 4.9×10^-2^ | 0.70* | 0.71* | 1.02 |
|  |  |  |  | ObIR+ | 6.2×10^-3^ | 0.82* | 0.79* | 0.95 |
| 1-Methylhistamine | 125.0953 | 0.57 | 1 | ObIR- | 1.1×10^-1^ | 0.67 | 0.76 | 1.14 |
|  |  |  |  | ObIR+ | 2.8×10^-3^ | 0.62* | 0.55* | 0.88 |
| Ornithine | 132.0899 | 0.58 | 1 | ObIR- | 6.9×10^-4^ | 0.76* | 0.73* | 0.95 |
|  |  |  |  | ObIR+ | 1.5×10^-4^ | 0.88 | 0.81* | 0.92 |
| Pipecolic acid | 129.0790 | 0.58 | 1 | ObIR- | 4.9×10^-2^ | 0.78* | 0.78* | 1.00 |
|  |  |  |  | ObIR+ | 8.8×10^-3^ | 0.88 | 0.82* | 0.94 |
| *Oxidative stress markers* | | | | | | | | |
| Pyroglutamic acid | 129.0426 | 0.94 | 1 | ObIR- | 2.8×10^-2^ | 0.57* | 0.52* | 0.91 |
|  |  |  |  | ObIR+ | 1.0×10^-2^ | 0.66* | 0.58* | 0.88 |
| Cysteinyl-glycine | 178.0412 | 0.96 | 1 | ObIR- | 2.1×10^-2^ | 0.82* | 0.77* | 0.93 |
|  |  |  |  | ObIR+ | 8.7×10^-4^ | 0.82* | 0.83* | 1.01 |
| Glutathione disulfide | 612.1520 | 1.22 | 1 | ObIR- | 4.9×10^-2^ | 0.57* | 0.52* | 0.92 |
|  |  |  |  | ObIR+ | 9.8×10^-3^ | 0.62* | 0.57* | 0.91 |
| *Nucleotide metabolism* | | | | | | | | |
| Inosine | 268.0808 | 1.55 | 1 | ObIR- | 4.9×10^-2^ | 1.24* | 1.40* | 1.12 |
|  |  |  |  | ObIR+ | 1.3×10^-2^ | 1.31 | 1.28* | 0.98 |
| *Sphingolipids* | | | | | | | | |
| CER(d18:1/14:0) | 509.4808 | 8.09 | 2 | ObIR- | 4.9×10^-2^ | 0.73* | 0.79 | 1.08 |
|  |  |  |  | ObIR+ | 1.1×10^-2^ | 0.81* | 0.77* | 0.95 |
| CER(d18:2/16:0) | 535.4964 | 8.19 | 2 | ObIR- | 4.9×10^-2^ | 0.76* | 0.77* | 1.01 |
|  |  |  |  | ObIR+ | 8.8×10^-3^ | 0.85* | 0.79* | 0.92 |
| SM(d18:1/15:0) | 688.5519 | 8.31 | 2 | ObIR- | 1.4×10^-2^ | 0.72* | 0.93 | 1.29* |
|  |  |  |  | ObIR+ | 5.4×10^-1^ | 0.93 | 0.96 | 1.03 |
| SM(d18:1/16:0) | 702.5676 | 8.44 | 2 | ObIR- | 2.4×10^-2^ | 0.82* | 0.93 | 1.13* |
|  |  |  |  | ObIR+ | 6.3×10^-1^ | 1.01 | 1.01 | 1.00 |
| *Other metabolites* | | | | | | | | |
| Hippuric acid | 179.0582 | 2.91 | 1 | ObIR- | 2.8×10^-3^ | 1.70* | 1.40* | 0.82 |
|  |  |  |  | ObIR+ | 7.2×10^-6^ | 1.93* | 1.61* | 0.84 |

**Table 5.** Differential erythroid metabolites after in vitro incubation with insulin in children with obesity and insulin resistance (ObIR+), children with obesity without insulin resistance (ObIR-), and healthy control children (CNT). * Indicates significant differences according to the post-hoc Fisher LSD test (p < 0.05).

| **Metabolite** | **Mass (Da)** | **RT (min)** | **MSI level** | **group** | **Fold changes** | | | |
| --- | --- | --- | --- | --- | --- | --- | --- | --- |
|  |  |  |  |  | **5 vs 0** | **12 vs 0** | **20 vs 0** | **150 vs 0** |
| *Glycolytic intermediates and related metabolites* | | | | | | | | |
| Lactic acid | 90.0317 | 0.95 | 1 | CNT | 1.00 | 1.14 | 1.39* | 1.24* |
|  |  |  |  | ObIR- | 0.96 | 1.07 | 1.12 | 1.21* |
|  |  |  |  | ObIR+ | 0.82 | 0.94 | 0.88 | 1.00 |
| *Branched chain amino acid catabolites* | | | | | | | | |
| 2,3-Dihydroxyisovaleric acid | 134.0579 | 1.37 | 1 | CNT | 3.08 | 3.84* | 2.95 | 2.27 |
|  |  |  |  | ObIR- | 0.96 | 1.00 | 1.17 | 1.47 |
|  |  |  |  | ObIR+ | 1.15 | 1.96 | 3.38* | 3.19* |
| Dihydroxyvaleric acid isomer | 134.0579 | 1.05 | 3 | CNT | 3.00* | 2.83 | 2.36 | 1.79 |
|  |  |  |  | ObIR- | 1.13 | 0.94 | 1.15 | 1.65 |
|  |  |  |  | ObIR+ | 1.74 | 1.61 | 3.15* | 3.09* |
| 3-Methyl-2-oxobutyric acid | 116.0473 | 1.95 | 1 | CNT | 1.91 | 1.88* | 1.83 | 1.65* |
|  |  |  |  | ObIR- | 0.72 | 0.92 | 0.83 | 0.88 |
|  |  |  |  | ObIR+ | 1.62 | 1.33 | 1.31 | 2.15* |
| 3-Methyl-2-oxovaleric acid | 130.0629 | 2.93 | 1 | CNT | 1.74* | 1.84* | 1.88* | 1.47* |
|  |  |  |  | ObIR- | 0.79 | 0.94 | 0.77 | 0.76 |
|  |  |  |  | ObIR+ | 1.30 | 1.22 | 1.24 | 1.59* |
| 4-Methyl-2-oxovaleric acid | 130.0629 | 3.09 | 1 | CNT | 1.80* | 1.89* | 1.86* | 1.45* |
|  |  |  |  | ObIR- | 0.84 | 1.21 | 0.77 | 0.82 |
|  |  |  |  | ObIR+ | 1.27 | 1.21 | 1.24 | 1.60* |
| 2-Hydroxy-4-methylvaleric acid | 132.0786 | 3.65 | 1 | CNT | 1.67* | 1.86* | 1.76* | 1.34 |
|  |  |  |  | ObIR- | 0.88 | 0.78 | 0.73 | 0.72 |
|  |  |  |  | ObIR+ | 1.25 | 1.19 | 1.26 | 1.68* |
| *Other amino acids* | | | | | | | | |
| L-Glutamic acid | 147.0532 | 0.69 | 1 | CNT | 1.04 | 1.19* | 1.21* | 1.19* |
|  |  |  |  | ObIR- | 1.03 | 1.11 | 1.11 | 1.17 |
|  |  |  |  | ObIR+ | 1.05 | 1.06 | 1.09 | 1.14 |
| L-Phenylalanine | 165.0790 | 2.01 | 1 | CNT | 0.89 | 1.29* | 1.18 | 1.09 |
|  |  |  |  | ObIR- | 1.17 | 1.19 | 0.95 | 0.95 |
|  |  |  |  | ObIR+ | 0.94 | 0.97 | 0.86 | 1.00 |
| N-Acetylaspartic acid | 175.0481 | 0.95 | 1 | CNT | 0.90 | 1.24 | 1.38* | 1.40* |
|  |  |  |  | ObIR- | 1.01 | 1.18 | 1.41* | 1.21* |
|  |  |  |  | ObIR+ | 1.18 | 1.12 | 1.59* | 1.26* |
| N-Acetylalanine | 131.0582 | 1.47 | 1 | CNT | 1.15 | 1.32 | 1.36* | 1.23 |
|  |  |  |  | ObIR- | 1.05 | 1.04 | 1.17 | 1.17 |
|  |  |  |  | ObIR+ | 0.96 | 0.96 | 1.04 | 1.11 |
| N-Acetylmethionine | 191.0616 | 2.70 | 1 | CNT | 1.22 | 1.42* | 1.69* | 1.58* |
|  |  |  |  | ObIR- | 0.92 | 0.87 | 1.14 | 1.07 |
|  |  |  |  | ObIR+ | 1.01 | 1.07 | 1.15 | 1.14 |
| Glutamyl-cysteine (or isomer) | 250.0623 | 0.94 | 3 | CNT | 1.77* | 1.67* | 1.59* | 1.31 |
|  |  |  |  | ObIR- | 0.89 | 1.10 | 1.14 | 0.92 |
|  |  |  |  | ObIR+ | 1.05 | 1.02 | 1.17 | 1.14 |
| Aspartyl-phenylalanine (or isomer) | 280.1059 | 1.86 | 3 | CNT | 0.74 | 1.33 | 1.43 | 1.62* |
|  |  |  |  | ObIR- | 0.88 | 1.03 | 1.15 | 0.99 |
|  |  |  |  | ObIR+ | 0.88 | 0.95 | 1.07 | 1.27 |
| Glutamyl-leucine (or isomer) | 260.1372 | 2.25 | 3 | CNT | 1.14 | 1.22* | 1.21* | 1.16 |
|  |  |  |  | ObIR- | 0.84 | 0.92 | 1.10 | 0.84 |
|  |  |  |  | ObIR+ | 1.11 | 1.13 | 1.04 | 1.36 |
| Glutamyl-tryptophan (or isomer) | 333.1325 | 2.27 | 3 | CNT | 1.03 | 1.43 | 1.81* | 1.75* |
|  |  |  |  | ObIR- | 0.76 | 0.79 | 1.19 | 0.94 |
|  |  |  |  | ObIR+ | 0.81 | 0.93 | 1.08 | 1.14 |
| Prolyl-leucine (or isomer) | 228.1474 | 2.29 | 3 | CNT | 1.35 | 1.45 | 1.86* | 2.03* |
|  |  |  |  | ObIR- | 0.92 | 0.84 | 1.11 | 0.72 |
|  |  |  |  | ObIR+ | 0.90 | 0.94 | 1.21 | 1.30 |
| *Oxidative stress markers* | | | | | | | | |
| Propenol glutathione (isomer 1) | 365.1257 | 1.37 | 3 | CNT | 0.72* | 0.67* | 0.66* | 0.64* |
|  |  |  |  | ObIR- | 0.92 | 0.83 | 0.97 | 0.99 |
|  |  |  |  | ObIR+ | 0.97 | 1.03 | 0.84 | 1.03 |
| Propenol glutathione (isomer 2) | 365.1257 | 1.47 | 3 | CNT | 0.67* | 0.63* | 0.83 | 0.76 |
|  |  |  |  | ObIR- | 0.96 | 0.82 | 0.91 | 0.94 |
|  |  |  |  | ObIR+ | 0.96 | 0.99 | 0.83 | 0.93 |
| 4-Hydroxyhexenal glutathione | 421.1519 | 2.21 | 2 | CNT | 0.53* | 0.61* | 0.56* | 0.59* |
|  |  |  |  | ObIR- | 1.09 | 0.98 | 0.90 | 1.23 |
|  |  |  |  | ObIR+ | 0.97 | 1.16 | 0.85 | 1.07 |
| Pentenal glutathione | 391.1413 | 2.45 | 2 | CNT | 0.73* | 0.82 | 0.78* | 0.78* |
|  |  |  |  | ObIR- | 1.07 | 1.10 | 1.02 | 1.04 |
|  |  |  |  | ObIR+ | 0.99 | 1.10 | 0.93 | 1.05 |
| 4-Oxohexenal glutathione | 419.1362 | 2.47 | 2 | CNT | 0.53* | 0.63* | 0.72 | 0.67 |
|  |  |  |  | ObIR- | 0.97 | 0.90 | 0.83 | 1.15 |
|  |  |  |  | ObIR+ | 0.95 | 1.10 | 0.84 | 1.00 |
| 4-Hexenol glutathione | 407.1726 | 2.81 | 2 | CNT | 0.54* | 0.44* | 0.62 | 0.68 |
|  |  |  |  | ObIR- | 0.97 | 0.94 | 0.97 | 1.42 |
|  |  |  |  | ObIR+ | 0.99 | 1.14 | 0.90 | 0.95 |
| 4-Hydroxynonenal glutathione | 463.1988 | 4.01 | 1 | CNT | 0.84 | 0.79 | 0.75 | 0.67* |
|  |  |  |  | ObIR- | 1.04 | 1.24 | 1.05 | 1.12 |
|  |  |  |  | ObIR+ | 0.96 | 1.13 | 0.88 | 1.02 |
| 4-Oxononenal glutathione | 461.1832 | 4.25 | 2 | CNT | 0.46* | 0.50* | 0.66 | 0.70 |
|  |  |  |  | ObIR- | 0.88 | 1.07 | 0.73 | 1.00 |
|  |  |  |  | ObIR+ | 0.80 | 1.05 | 0.81 | 0.99 |
| Nonenol glutathione (isomer 1) | 449.2196 | 4.41 | 3 | CNT | 0.60* | 0.67 | 0.74 | 0.70 |
|  |  |  |  | ObIR- | 0.97 | 0.98 | 0.83 | 1.13 |
|  |  |  |  | ObIR+ | 0.90 | 1.10 | 0.93 | 0.92 |
| Nonenol glutathione (isomer 1) | 449.2196 | 4.63 | 3 | CNT | 0.59* | 0.66* | 0.75* | 0.67* |
|  |  |  |  | ObIR- | 0.90 | 1.04 | 0.87 | 1.10 |
|  |  |  |  | ObIR+ | 1.25 | 1.40 | 1.02 | 1.23 |
| Dodecadienal glutathione | 487.2352 | 4.68 | 3 | CNT | 0.59* | 0.64* | 0.65* | 0.63* |
|  |  |  |  | ObIR- | 0.94 | 1.01 | 0.86 | 1.09 |
|  |  |  |  | ObIR+ | 0.90 | 1.00 | 0.79 | 0.91 |
| Hydroxydecenal glutathione | 477.2145 | 4.83 | 3 | CNT | 0.58 | 0.65* | 0.70* | 0.70* |
|  |  |  |  | ObIR- | 0.99 | 0.93 | 0.81 | 1.12 |
|  |  |  |  | ObIR+ | 1.03 | 1.14 | 0.91 | 1.01 |
| *Dicarboxylic acids* | | | | | | | | |
| 3-Hydroxy-3-methylglutaric acid | 162.0528 | 1.40 | 1 | CNT | 0.79 | 0.93 | 0.91 | 0.90 |
|  |  |  |  | ObIR- | 1.33 | 1.40* | 1.48* | 1.53* |
|  |  |  |  | ObIR+ | 0.96 | 0.99 | 1.04 | 1.00 |
| Methylsuccinic acid | 132.0423 | 2.18 | 1 | CNT | 0.96 | 0.84 | 0.68 | 1.01 |
|  |  |  |  | ObIR- | 1.43 | 1.37 | 1.52* | 2.62* |
|  |  |  |  | ObIR+ | 0.78 | 0.70 | 0.85 | 1.03 |
| 3-Methylglutaric acid | 146.0579 | 2.55 | 1 | CNT | 1.32 | 1.07 | 1.24 | 1.34 |
|  |  |  |  | ObIR- | 1.57 | 2.18* | 1.29 | 1.51 |
|  |  |  |  | ObIR+ | 0.93 | 1.26 | 1.38 | 1.35 |
| Pimelic acid | 160.0735 | 3.24 | 1 | CNT | 1.37* | 1.06 | 1.18 | 1.15 |
|  |  |  |  | ObIR- | 1.21 | 1.77* | 1.35 | 1.62* |
|  |  |  |  | ObIR+ | 0.98 | 1.00 | 1.06 | 1.14 |
| Suberic acid | 174.0892 | 3.88 | 1 | CNT | 1.22 | 1.01 | 1.03 | 1.08 |
|  |  |  |  | ObIR- | 1.27 | 1.35 | 1.08 | 1.21* |
|  |  |  |  | ObIR+ | 1.02 | 1.06 | 1.08 | 1.10 |
| *Phospholipids* | | | | | | | | |
| LPC(20:4) | 543.3325 | 6.69 | 3 | CNT | 0.93 | 0.86* | 0.89* | 0.92 |
|  |  |  |  | ObIR- | 0.92 | 0.88* | 0.84* | 0.90 |
|  |  |  |  | ObIR+ | 1.01 | 1.02 | 1.07 | 0.98 |
| LPE(22:6) | 525.2855 | 6.70 | 3 | CNT | 0.72* | 0.78 | 0.75* | 0.74* |
|  |  |  |  | ObIR- | 1.07 | 1.01 | 0.97 | 1.01 |
|  |  |  |  | ObIR+ | 1.00 | 1.07 | 0.94 | 1.04 |
| LPE(18:2) | 477.2855 | 6.71 | 3 | CNT | 0.73* | 0.71* | 0.82 | 0.82 |
|  |  |  |  | ObIR- | 1.16 | 0.91 | 0.82 | 0.83 |
|  |  |  |  | ObIR+ | 0.94 | 0.99 | 1.00 | 1.10 |
| LPE(22:5) | 527.3012 | 6.77 | 3 | CNT | 0.71* | 0.76* | 0.73* | 0.71* |
|  |  |  |  | ObIR- | 1.04 | 0.99 | 0.95 | 0.99 |
|  |  |  |  | ObIR+ | 1.06 | 1.13 | 0.98 | 1.10 |
| LPE(20:3) | 503.3012 | 6.80 | 3 | CNT | 0.72* | 0.74* | 0.73 | 0.66* |
|  |  |  |  | ObIR- | 0.97 | 0.94 | 0.86 | 0.98 |
|  |  |  |  | ObIR+ | 0.95 | 1.03 | 0.90 | 0.99 |
| LPC(18:2) | 519.3325 | 6.87 | 3 | CNT | 0.80 | 0.80 | 0.69* | 0.73* |
|  |  |  |  | ObIR- | 1.24 | 1.28 | 0.95 | 1.44 |
|  |  |  |  | ObIR+ | 1.04 | 0.96 | 0.89 | 0.99 |
| LPE(P-16:0) | 437.2906 | 6.97 | 3 | CNT | 0.76* | 0.72* | 0.76 | 0.67* |
|  |  |  |  | ObIR- | 0.97 | 0.99 | 0.76 | 0.86 |
|  |  |  |  | ObIR+ | 0.89 | 1.02 | 0.85 | 0.98 |
| PC(16:0/22:6) | 805.5622 | 8.72 | 3 | CNT | 0.99 | 0.95 | 0.87* | 1.01 |
|  |  |  |  | ObIR- | 0.84 | 0.97 | 0.98 | 0.91 |
|  |  |  |  | ObIR+ | 1.04 | 0.99 | 1.05 | 1.09 |
| *Sphingolipids* | | | | | | | | |
| SM(d16:1/18:1) | 700.5519 | 8.18 | 2 | CNT | 0.78* | 0.95 | 0.85 | 0.93 |
|  |  |  |  | ObIR- | 0.92 | 1.08 | 0.88 | 0.85 |
|  |  |  |  | ObIR+ | 1.03 | 1.00 | 0.95 | 1.02 |
| SM(d18:1/18:0) | 730.5989 | 8.80 | 2 | CNT | 0.68* | 0.88 | 0.83 | 0.71* |
|  |  |  |  | ObIR- | 1.05 | 0.80 | 1.00 | 1.14 |
|  |  |  |  | ObIR+ | 0.95 | 0.87 | 0.88 | 1.08 |
| SM(d18:1/24:2) | 810.6615 | 9.73 | 2 | CNT | 0.84* | 0.82* | 0.89 | 0.88 |
|  |  |  |  | ObIR- | 0.95 | 0.96 | 1.03 | 0.95 |
|  |  |  |  | ObIR+ | 1.01 | 1.09 | 1.04 | 0.98 |


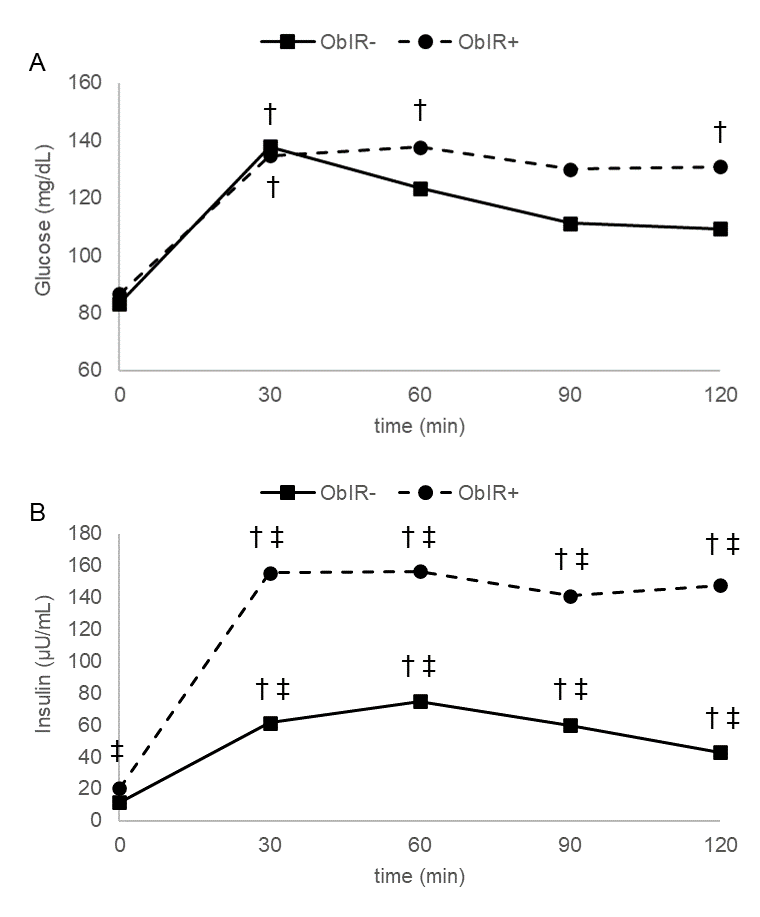


**Figure S1.** Time-dependent changes in blood glucose (A) and insulin (B) along the oral glucose tolerance test. ^†^ Denotes significant differences with respect to baseline determinations, ^‡^ denotes significant differences between ObIR+ and ObIR- groups.
